# Beyond pathogens: the intriguing genetic legacy of endogenous retroviruses in host physiology

**DOI:** 10.3389/fcimb.2024.1379962

**Published:** 2024-04-09

**Authors:** Amanda Lopes da Silva, Bruno Luiz Miranda Guedes, Samuel Nascimento Santos, Giovanna Francisco Correa, Ariane Nardy, Luiz Henrique da Silva Nali, Andre Luis Lacerda Bachi, Camila Malta Romano

**Affiliations:** ^1^ Instituto de Medicina Tropical de São Paulo, Faculdade de Medicina da Universidade de São Paulo, São Paulo, Brazil; ^2^ UNISA Research Center, Universidade Santo Amaro, Post-Graduation in Health Sciences, São Paulo, Brazil; ^3^ Hospital das Clinicas HCFMUSP, Faculdade de Medicina, Universidade de Sao Paulo, Sao Paulo, Brazil

**Keywords:** endogenous retroviruses, genetic regulation, inflammaging, transactivation, inflammatory diseases

## Abstract

The notion that viruses played a crucial role in the evolution of life is not a new concept. However, more recent insights suggest that this perception might be even more expansive, highlighting the ongoing impact of viruses on host evolution. Endogenous retroviruses (ERVs) are considered genomic remnants of ancient viral infections acquired throughout vertebrate evolution. Their exogenous counterparts once infected the host’s germline cells, eventually leading to the permanent endogenization of their respective proviruses. The success of ERV colonization is evident so that it constitutes 8% of the human genome. Emerging genomic studies indicate that endogenous retroviruses are not merely remnants of past infections but rather play a corollary role, despite not fully understood, in host genetic regulation. This review presents some evidence supporting the crucial role of endogenous retroviruses in regulating host genetics. We explore the involvement of human ERVs (HERVs) in key physiological processes, from their precise and orchestrated activities during cellular differentiation and pluripotency to their contributions to aging and cellular senescence. Additionally, we discuss the costs associated with hosting a substantial amount of preserved viral genetic material.

## 1 Background

Viruses are traditionally known as parasitic and pathogenic agents that can infect all living beings. However, the advent of genetic sequencing has been placing the viruses in a much better context. Retroviruses, in particular, contain all essential genes for integrating their RNA genome into the host genome. Following integration, the retroviral locus is named provirus, which can either express retroviral proteins or serve as the template for new retroviral genome synthesis (Nisole and Saïb, 2004). Usually, retroviruses infect somatic cells, but if a germ cell becomes infected, the provirus may be transmitted on to subsequent generations. Across generations of all vertebrates, including humans and their primate ancestors, successive waves of retroviral infections have introduced proviruses into our germline cells, and today, they are known as Human Endogenous Retroviruses (HERVs) (Nisole and Saïb, 2004; Jern and Coffin, 2008).

As classical retroviruses, the genomic structure of ERVs comprises gag, pro-pol, and envelope genes, flanked by two long terminal repeats (LTRs). Several ERV families have integrated into the ancestral host germ line cells and proliferated due to active replication and retroposition, and now account for ~8% of the human genome (Nurk et al., 2022). While some HERVs (human ERV) exhibit transcriptional activity, the majority of retroviral sequences in the human genome have been compromised by mutations or successive insertion and/or deletions and recombination (Bannert and Kurth, 2006; Vargiu et al., 2016). This is likely a consequence of the detrimental impact of the active retrotransposition on the host genome, resulting in approximately 85-90% of ERV loci being represented by solo-LTR. Indeed, most HERVs lack intact open reading frames, and no autonomously replicating HERV has been identified. Consequently, HERVs are generally perceived as non-functional (Vargiu et al., 2016). However, as we will discuss later in this review, specific HERV genes or their LTRs persist in an active state and play a role in the host genetic network (Jern and Coffin, 2008).

The International Committee on the Taxonomy of Viruses (ICTV) classifies ERVs according to the similarity and phylogenetic relationship to exogenous retroviruses. Class I ERVs are those that cluster with *Gammaretrovirus* and *Epsilonretrovirus*, Class II ERVs cluster with *Alpharetrovirus, Betaretrovirus, Deltaretrovirus*, and *Lentivirus*, and Class III ERVs are closer to foamy viruses and ERV-L (Coffin et al., 2021).

Traditionally, the names of the HERV families have been denoted by a letter, based on the specific type of the amino acid of human tRNA that binds to the primer binding site (PBS) during the reverse transcription process. For instance, HERV elements that utilize a Lysine tRNA are named HERV-K. Some groups were also sporadically named concerning a particular amino acid motif (e.g., HERV-FRD). However, the modern taxonomic classification of HERV is based on phylogenetic approaches of the conserved *pol* gene or, in some cases, on the LTR (Jern et al., 2005).

In the human genome, HERVs are represented by more than 717.7 individual elements, classified into 30 families. The largest family is HERV-H, which integrated into the primate genome before the divergence of New and Old-World Monkeys (de Parseval et al., 2001). HERV-H has roughly 1000 elements (complete or near complete) and an even greater number of solo-LTR (Guliyev et al., 2013).

The HERV-K family represents the most recent integration into the genomes of Old-World primates. It is also very large, with at least 11 independent introductions in the primate ancestral genome. The elements within the HERV-K family have been classified based on sequence similarities to Mouse Mammary tumor viruses, named HML-(1 to 11) from human MMTV- like (Subramanian et al., 2011). Among them, the HML-2 subfamily is the most recent and best preserved one, with 89 complete elements and ~1000 solo LTR. Most studies on HML elements are focused on HML-2, which, interestingly, harbors several polymorphic sites in the human genome, as it remained transcriptionally active until very recently in evolution (Belshaw et al., 2005).

The HERV-W, a gammaretrovirus-like also integrated into the host genome after the divergence of the New and Old-World primates, though fewer complete proviruses (LTR-gag-pro-pol-env-LTR) remained in our genome in comparison to HERV-K (Tristem, 2000; Grandi et al., 2016).

HERV-W is classified in subgroup 1 and subgroup 2 according to their LTRs, and almost 70% of the 213 elements of this family belong to subgroup 1 (Grandi et al., 2016). HERV-W is also an extremely active family, and the best-known example is the functional envelope gene placed in chromosome 7q21.1. Syncytin-1, a cell-cell fusion protein is encoded by this retroviral gene, also known as ERVWE-1, which was co-opted by the host genome and is actively expressed during the trophoblast formation during pregnancy (Mi et al., 2000; Grandi et al., 2016).

Recent epigenomic studies have brought to light ERVs as an unexpectedly significant source of cell type-specific regulatory elements. These encompass promoters, enhancers, chromatin boundary elements, and regulatory RNAs. Among the vast array of proviruses and solo-LTRs, approximately 320,000 appear to hold active transcription binding sites, implying their involvement in regulating various host genes after a process of domestication (Garazha et al., 2015). While most elements remain transcriptionally silent under process as methylation and histone modification (Groh and Schotta, 2017), several elements can be re-activated by multiple environmental and intrinsic factors such as hormones, cellular co-factors, aging-associated processes, epigenetic drugs, radiation, chemicals and also, by exogenous viruses (Nellåker et al., 2006; Contreras-Galindo et al., 2007b; Vincendeau et al., 2015; Geis and Goff, 2020; Hurme and Pawelec, 2021).

**Figure 1:**
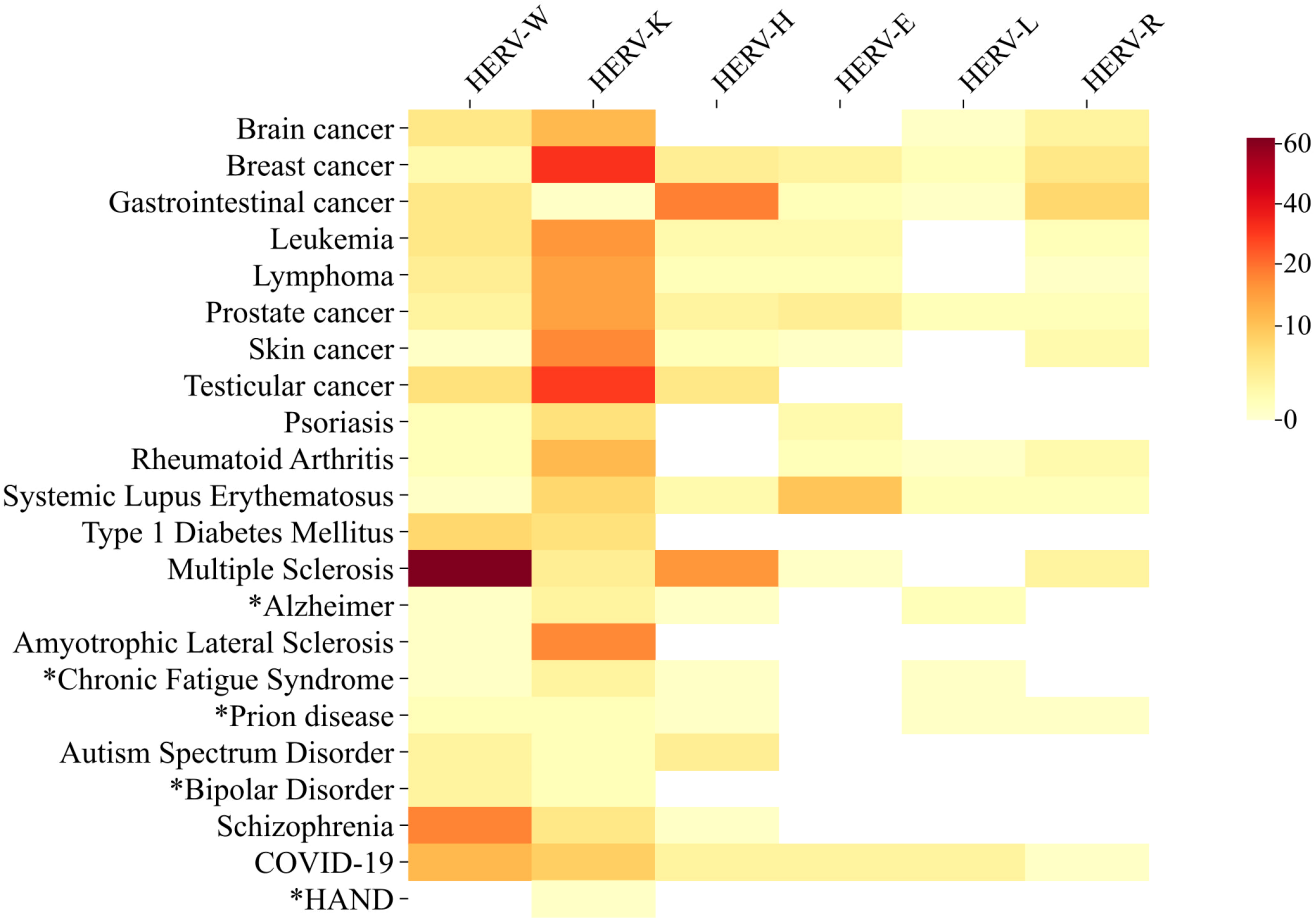
Heat Map of Studies Investigating Involvement of Main HERV Families with Relevant Diseases and Disorders (DD). The heat map illustrates the number of public available studies (1979-2023) investigating the association between the main HERV families and relevant diseases. Darker colors represent a higher number of published articles, while lighter colors indicate fewer publications. The colors do not necessarily reflect a positive correlation between HERV activity and disease, as studies describing downregulation or absence of correlation were also included in the analysis. **Supplementary File 1** include the list of the articles used to build this figure. Data on cancer/HERV published up to October 2023 were retrieved from CancerHERVdb (available at https://erikstricker.shinyapps.io/cancerHERVdb/). footnotes. (*) Diseases with < 10 studies including the *Human Endogenous Retroviruses* subject. HAND- HIV-associated neurocognitive disorder. COVID-19 - Coronavirus diseases 2019.

Notably, HERVs became famous primarily due to their suspected involvement in diseases (see **Figure 1**), when retroviral particles were initially observed in testis tumor cells and patients with Multiple Sclerosis (Bronson et al., 1979; Perron et al., 1997). In fact, the association between HERVs and the pathogenesis of Multiple Sclerosis stands out as one of the most thoroughly investigated links. One of the proposed theories suggests that molecular mimicry between HERV-W/envelope and myelin proteins may trigger nonspecific responses against myelin (Olival et al., 2013; Ramasamy et al., 2017; de Luca et al., 2019), leading to its degradation. Over the last decades, the detection of HERV-derived transcripts, proteins, as well as anti-HERV antibodies, and viral particles in various pathological conditions, has brought HERVs unfortunate notoriety (Contreras-Galindo et al., 2007b; Perzova et al., 2013; Volkman and Stetson, 2014; Horssen et al., 2016).

**Figure 2:**
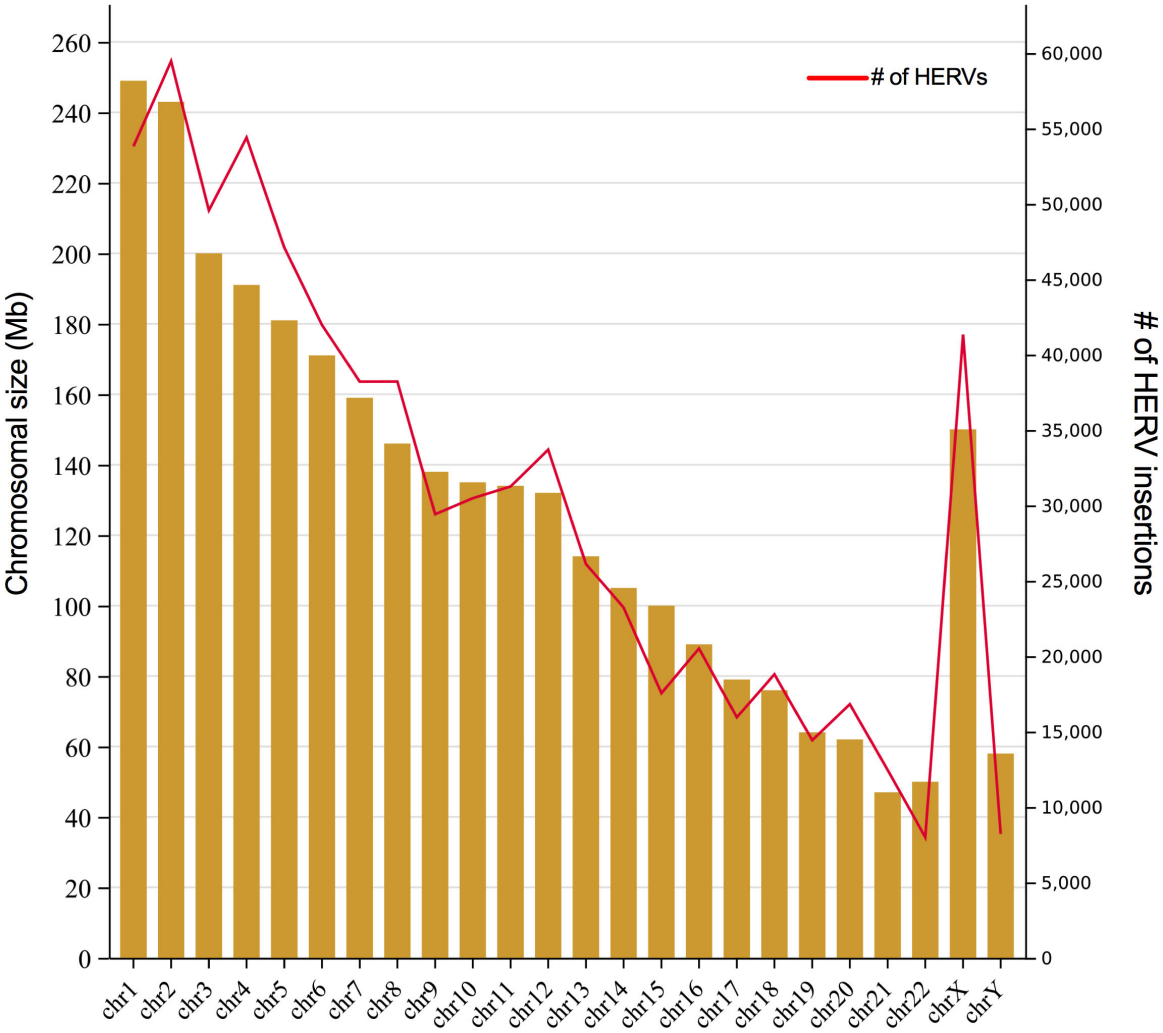
Number of HERVs per human chromosome. The graph shows the size of each human chromosome in Mb (left axis) and the number of HERVs insertion (partial and complete) per chromosomes (right axis). Retrovirus integration data was obtained from HERVd.

Yet, despite HERVs having been implicated in many pathological processes, it seems a paradox that we have nearly 20-fold more retroviral sequences than there are human genes in our genome. **Figure 2** shows the number of LTR-elements (provirus and LTR) mapped in each chromosome according to HERVd (Paces, 2004) as well as chromosome size. Despite we did not estimate the ratio of integration per chromosomal size, apparently the HERVs are evenly dispersed through them, with some notable exceptions, as repeatedly observed for the chromosome Y and 19 (Kim et al., 2004; Subramanian et al., 2011). In general, the GC-content, gene richness and recombination rate correlate with HERVs density in each chromosome (Katzourakis et al., 2007). But, the final fixation of solo LTR or complete elements ultimately depends on the impact on the nearby genes.

Several studies have shed light on the pivotal contribution of LTRs in promoting or enhancing the expression of several genes in humans. Particularly those LTRs integrated near genes, appear to have been repurposed as regulatory elements, as evidenced by strong purifying selection (Lowe et al., 2007). Therefore, it is now evident that many of these elements were co-opted through evolution, and today, play a constructive role in normal human physiology (**Table 1**).

**Table 1:** HERV families and host genetic events.

HERV-involved events	HERV families	Methods	References
Host genetic regulation	HERV-K; HERV-E; HERV-W and HERV-H; ERV-9.	RTPCR; Sequencing; Protein detection	(Bergallo et al., 2018; Durnaoglu et al., 2021; Hashimoto et al., 2021; Zhang et al., 2022)
Pluripotency/ embryogenesis	HERV-K: LTR5HS; LTR3B, LTR14B; HERV-H: LTR7B and LTR7Y; HERV-W; HERV-L: MLT2A1; ERV-9: LTR12C.	Cell culture; Sanger sequencing; siRNA knockdown; bioinformatics.	(Noorali et al., 2009; Kämmerer et al., 2011; Macfarlan et al., 2012; Santoni et al., 2012; Dunn et al., 2014; Lu et al., 2014; Ohnuki et al., 2014; Wang et al., 2014; Göke et al., 2015; Grow et al., 2015; Kannan et al., 2015; Soygur and Moore, 2016; Hendrickson et al., 2017; Vastenhouw et al., 2019)
Placentation	HERV-K; HERV-W: Syncytin-1; HERV-FRD: Syncytin-2.	RT PCR; Immunohistochemical; Cell culture.	(Noorali et al., 2009; Kämmerer et al., 2011; Grandi et al., 2016; Soygur and Moore, 2016)
Overexpression in cancer	HERV-W;HERV-P; HERV-R; HERV-H: LTR-7; LTR7Y; HERV-L: LTR2B; HERV-FRD; HERV-E.	Cell culture; RNA-seq; RT PCR; Literature review.	(Bergallo et al., 2018; Deng et al., 2019; Siebenthall et al., 2019; Zhang et al., 2022)
Retroviral – transactivation	HIV: HERV-K: HML2; HERV-E; HERV-T; ERV-9. HTLV: HERV-K	Immunoblotting; Cloning/sequencing; RT PCR.	(Contreras-Galindo et al., 2006; Toufaily et al., 2011; Perzova et al., 2013; Dai et al., 2018; Chen et al., 2019)
Non-retro viral transactivation	SARS-CoV-2: HERV-W env; Herpesviruses in general: HERV-K; HERV-W; HERV-H	RT PCR; ME.	(Poole et al., 2006; Li et al., 2014; Chen et al., 2019; Charvet et al., 2023)
Neurological/ autoimmune diseases	ALS and MS- HERV-K: HML2; Alzheimer disease: HERK-K: LTR5H. Prion disease and MS: HERV-W; HERV-L; HERV-FRD; ERV-9. Rheumatic disease: HERV-K; HERV-H; HERV-WE1; HERV-WE2.	qRT PCR; Sequencing; Northern blot; RNAseq	(Poole et al., 2006; Jeong et al., 2010; Douville et al., 2011; Bendiksen et al., 2014; Gruchot et al., 2019; Ovejero et al., 2020; Li et al., 2022; Nali et al., 2022)
Neurological/ psychiatric diseases	HERV-WK10; HERV-W *gag*; HERV-W *env.*	RT-PCR	(Slokar and Hasler, 2015; Aftab et al., 2016; Gruchot et al., 2019; Gruchot et al., 2023a)
Aging	HERV-K: HML2; HERV-W.	RT-PCR; Illumina HiSeq	(Johnston et al., 2001; Rolland et al., 2005; Rolland et al., 2006; Mameli et al., 2007; Balestrieri et al., 2015; Nevalainen et al., 2018)

We are now aware of the pivotal role of HERVs in regulating the homeostasis of their hosts. This review will explore the involvement of HERVs in some key events, as well as embryogenesis, inflammation, and aging. We also discuss how the dysregulation of these elements might be associated with cancer, neurodegenerative, and autoimmune diseases. Finally, we will address certain controversial findings surrounding the reactivation of HERVs in response to exogenous virus infections.

## 2 HERVs in embryogenesis and pluripotency

While numerous genes contribute to pluripotency, accumulating evidence has demonstrated that transposable elements, especially endogenous retroviruses, participate in the pluripotency genetic network, as largely demonstrated in both human and mouse models (Babu et al., 2004). The meticulous control of ERV expression is intricately managed during embryonic development, particularly through methylation and histone modification (Groh and Schotta, 2017). But there are two occasions that an epigenetic reset occurs, temporarily altering the DNA and histone methylation status: after fertilization, and during gametogenesis (Voon and Gibbons, 2016). These periods of global demethylation facilitate a broad HERV activity since transcriptional repression is lifted. However, the HERV expression is not random or uncontrolled. Highly orchestrated control, alternating between overexpression and decreased activity of specific families and individual elements, suggests a very specific role for HERVs during embryogenesis (Göke et al., 2015; Voon and Gibbons, 2016).

At the developmental stage, both parental genomes of the zygote must be reprogrammed to accomplish the transition from a terminally differentiated state to a totipotency state. The embryonic development is regulated from the transition of the oocyte to the embryo at the early stage of embryogenesis, during a process termed maternal‐to‐zygotic transition (MZT) (Vastenhouw et al., 2019). During MZT, the maternal components are degraded, giving space to the zygotic genome activation (ZGA). The ZGA occurs gradually after fertilization and is a critical step during the initial stages of cell cleavage during embryogenesis. DUX is a family of transcription factors shared by humans and mice. Dux in mice and its ortholog in humans, DUX4 activates several genes during ZGA, including endogenous retroviruses. In murine models, MuERV-L is activated during this phase, representing up to 3% of all mRNA, as is also observed for HERV-L in humans (Macfarlan et al., 2012; Hendrickson et al., 2017).

Accumulating data have demonstrated that HERVs in general, including members from the K, W, L, and H families, have been implicated in human stem cell identity and embryonic development (Macfarlan et al., 2012; Göke et al., 2015; Hendrickson et al., 2017; Vastenhouw et al., 2019). Additionally, HERVs exhibit expression patterns specific to different developmental stages and have either been demonstrated or predicted to be associated with lineage specification.

Goke et al., 2015 (Göke et al., 2015) identified that the LTR families displaying stage-specific expression in early embryos are not necessarily active in adulthood. They could trace a timeline of HERV or HERV-derived promoters during the early embryogenesis, when LTR3B and LTR14B are active from oocyte to four-cell, followed by LTR12C (from zygote to eight-cell), MLT2A1 and THE1A, that together with HERV-L, are expressed at the eight-cell stage, and LTR5_Hs, related to HERV-K, is more active during the morula stage. During the blastocyst stage, LTR7 and LTR7Y from HERV-H reach the activation peak (Göke et al., 2015). Critically, only a few elements present transcription initiating out of the promoter region, demonstrating that the majority of ERV-derived transcripts are indeed produced and regulated by their own LTR.

The HERV-H is one the most active retroviral families during the embryonic stage, accounting for 2% of all RNA transcripts in human embryonic stem cells (hESCs), therefore, providing a precise marker for pluripotency in human cells (Santoni et al., 2012; Wang et al., 2014). This activity is very likely due to its well-conserved LTRs (LTR7/HERV-H), that can be activated by multiple pluripotent transcription factors (TF) (Dunn et al., 2014). Around 80% of the highly expressed LTR7 harbor key TF binding sites related to pluripotency, such as OCT3/4, SOX-2, and NANOG (Ohnuki et al., 2014).

Several roles in differentiation and pluripotency have been assigned to HERV-H including harboring functional enhancers, super-enhancers, and alternative promoters, and the synthesis of long noncoding RNAs (lncRNAs) (Lu et al., 2014; Göke et al., 2015). It is estimated that 10% of HERV-H transcripts are lncRNAs (Kannan et al., 2015). Long non-coding RNA is a class of RNAs longer than 200 nucleotides that display several functions. Recent evidence also points to a fundamental role of lncRNA as an epigenetic regulator of stem cell pluripotency and specific lineage commitment (Mercer et al., 2009).

Some modulatory effects on the homeostasis of the human embryonic stem cell (hESC) were confirmed by knockdown experiments, where silencing HERV-H resulted in the loss of pluripotency of hESC and also impaired reprogramming of somatic cells to induced pluripotent stem cells (iPSC) (Lu et al., 2014; Wang et al., 2014). More recently, it was demonstrated that almost all HERV-H transcripts in hESCs belong to one of the youngest HERV-H subfamilies (10-14 Mya), LTR7up (Carter et al., 2022).

HERV-K mRNAs and proteins are also detected during typical human embryogenesis. The transcription of HERV-K, along with its accessory Rec protein, begins at the 8-cell stage, extending through epiblast cells in preimplantation embryos until the formation of embryonic stem cells, where the production of HERV-K mRNA ceases. Remarkably, the significance of HERV-K at the human blastocyst stage is marked by the detection of the capsid protein (gag) from HERV-K and by the presence of virus-like particles resembling Class-II retroviral particles (Grow et al., 2015). Later on, HERV-K envelope expression is detected again in placental tissue, more specifically in villous cytotrophoblast (VT) and extravillous cytotrophoblast (EVT) cells (Kämmerer et al., 2011), but not in syncytiotrophoblast, where only Syncytin protein is detected.

The expression of Syncytin-1, an HERV-W-derived protein in trophoblasts (alongside HERV-FRD or Syncytin-2, an even older endogenous retroviral co-option) is indispensable for cell-cell fusion, facilitating the formation of syncytiotrophoblast during the early stages of pregnancy (Mi et al., 2000). The trophoblast tissue is essential for invasive placental development and the prevention of immune rejection of the fetus at the fetus-maternal interface. According to immunolocalization studies, Syncytin-1 expression is a prerequisite for embryo implantation (Noorali et al., 2009; Soygur and Moore, 2016). Due to the fusogenic role of this protein, Syncytin-1 is also thought to be involved in fertilization, where it would contribute to the fusion of gametes since sperm cells express Syncytin-1 on the cell surface whereas oocytes express the syncytin-1 receptor SLC1A5 (Soygur and Sati, 2016).

## 3 Inflammaging, cellular senescence, and HERVs

Inflammation is a vital, elementary, and evolutionarily conserved biological response of different cell types, both immune and non-immune cells, needed to not only protect the host but also promote tissue repair and recovery after the occurrence of a cell/tissue injury triggered by several agents, such as damaged cells, pathogens, irradiation, and toxins (Chen et al., 2017; Furman et al., 2019).

During acute inflammation, the interplay of cellular and molecular events is crucial to limit excessive inflammatory activity, mitigating potential harm and aiding in the elimination of the causative agent. Therefore, acute inflammation has to persist until the threat or injury is resolved, after which it naturally subsides. Uncontrolled acute inflammation, when resolution is hindered, can lead to chronicity, increasing the risk of various chronic inflammatory diseases (Zhou et al., 2016; Chen et al., 2017; Furman et al., 2019).

The term “inflammaging” was coined in 2000 and translates as a phenomenon characterized by a sterile, systemic, chronic, and subclinical low-grade inflammation associated with aging (Fulop et al., 2018). Inflammaging is likely involved in the development and progression of most of the diseases common in the older population, particularly chronic inflammatory diseases (Fülöp et al., 2016; Franceschi et al., 2018). In aging, irrespective of gender, chronic immune activation occurs due to environmental factors and cellular senescence, contributing to inflammaging (Bektas et al., 2017). This aging-related immune dysfunction leads to the release of pro-inflammatory mediators into the bloodstream, characterizing inflammaging even in the absence of active diseases (Frasca and Blomberg, 2016). Elevated systemic levels of cytokines such as IL-1β, IL-6, and TNF-α not only perpetuate and intensify inflammaging but are also linked to age-related diseases, exerting a detrimental influence on healthspan (Franceschi et al., 2017; Fulop et al., 2018; Jia et al., 2022; Fulop et al., 2023).

Current data has pointed out that both aging-associated alterations and a chronic pro-inflammatory state can impact the activation of HERVs (Morris et al., 2019; Hurme and Pawelec, 2021). And, also the opposite, HERV reactivation can increase the risk of developing aging-related diseases, particularly neurodegenerative and autoimmune diseases (Compston and Coles, 2008; Mao et al., 2021). Based on these pieces of information, it is reasonable to suggest that the vicious circle between inflammation and HERV expression can become a trigger and a sustainer of favorable soil to clinical manifestations of age-related diseases. It was demonstrated that HERV-W products engage with Toll-like receptors (TLRs), notably TLR4 and CD14, triggering an inflammatory response with the secretion of cytokines (IL-1β, IL-6, TNF-α), potentially linked to age-related diseases (Rolland et al., 2005; Rolland et al., 2006). In this “interactive looping”, TNF-α transactivates different families of HERVs, via TNF-α receptor signaling and subsequent activation of NF-kB that also promotes the expression of HERVs, potentially impacting the incidence of aging-related diseases (Johnston et al., 2001; Mameli et al., 2007; Balestrieri et al., 2015).

Supporting the implication of Human Endogenous Retroviruses (HERVs) in the aging process, there are notable changes in expression levels throughout the lifespan (Cardelli, 2018). This expression pattern is also contingent on the specific HERV family under consideration. For instance, the expression of HERV-K and HERV-W in infants (< 1 year) is consistently present but not significantly elevated (Nali et al., 2017), in contrast to the increased expression observed in adults (Balestrieri et al., 2015). Notably, the global HERV expression, particularly of the HERV-W family, peaks in older adults (> 60 years old). Conversely, HERV-H, known for its close association with cellular differentiation, reaches its expression peak in children up to 4 years old. Subsequently, its expression remains at a basal level throughout adulthood, experiencing an upturn after the age of 60 (Balestrieri et al., 2015).

At this point, it is utmost of importance to highlight the well-known relation between HERV activity and the risk of developing aging-related diseases, particularly neurodegenerative and autoimmune diseases, in which chronic inflammation is also crucial for their pathogenesis (Compston and Coles, 2008; Mao et al., 2021). Of interest, the ability of HERVs to influence the immune responses, particularly innate immunity, seems to drive an abnormal and exacerbated inflammatory reaction that can contribute to fuel chronic inflammation (Hurst and Magiorkinis, 2015).

Epigenetic aspects play a key role in phenotypic aging-related changes and their causal mechanisms, contributing not only to cellular senescence but also to the development of the senescence-associated secretory phenotype (SASP) (Cardelli, 2018; Hurme and Pawelec, 2021). Aged tissues commonly accumulate senescent cells, losing proliferative and functional capacities, exhibiting apoptosis resistance, and producing SASP factors that ultimately fuel inflammation (Zhou et al., 2023). The insufficient clearance of senescent cells leads to systemic inflammation through higher expression of pro-inflammatory cytokines via SASP. These effects may potentially increase the risk of developing age-related diseases (LeBrasseur et al., 2015).

Based on the presented information, cellular senescence emerges as a major contributing factor to aging, with recent studies exploring the role of HERVs in this context. *In vitro* senescence models revealed increased expression of retroelements, particularly the HERV-K (HML-2) family, in prematurely aged human mesenchymal progenitor cells from progeroid syndrome patients compared to healthy cells. Additionally, elevated HERV-K (HML-2) env protein levels were observed in senescent cell culture supernatants. Notably, the introduction of these products to younger cells induced rapid senescence, likely due to loss of epigenetic control, but treatment with anti-Env antibodies blocked this senescent effect (Hurme and Pawelec, 2021; Liu et al., 2023). The study also demonstrated that HERV-K DNA accumulation in the cytoplasm of senescent cells activates innate immunity and SASP, but its depletion was capable of mitigating cellular senescence by attenuating SASP and immune responses. Conversely, HERV activation in young cells induced immune responses and cytokine secretion by SASP (Liu et al., 2023). These findings suggest that HERV directly impacts aging, as well as this intriguing influence could lead to a wider and unifying understanding of molecular regulators involved in the spreading of cellular senescence.

The impact of endogenous retroviruses on host genetic regulation is undeniable. However, the preservation of these elements has not occurred without consequences. Increasing evidence has associated the dysregulation of HERVs with tumorigenesis, inflammatory and neurodegenerative diseases, as abovementioned. Multiple sclerosis emerges as one of the diseases wherein the expression of endogenous retroviruses has been thoroughly investigated. Hypotheses, such as molecular mimicry or inflammatory triggers arising from envelope protein expression, are subjects of extensive discussion (Ramasamy et al., 2017; de Luca et al., 2019). Yet, other less-explored diseases in terms of HERV expression also seem to exhibit connections with the dysregulation of these elements.

Cellular senescence, an initial defense against cancer during early life, is now considered a fundamental aging process that contributes to developing aging traits and age-related diseases in later stages of life, such as tumorigeneses. And how much HERVs are implicated in malignancies is also a matter of debate.

## 4 Endogenous retroviruses in malignancies

The initial studies linking HERVs to cancer development date back to the early 1970s, when the presence of reverse transcriptase (RT) activity and viral particles within cancer cells were first described (Wang Y. et al., 1995; Feller and Chopra, 1968; Sarngadharan et al., 1972). Little further, it was found that human breast cancer cells expressed RNA that was very similar to the well-known Mouse Mammary Tumor Virus (MMTV) RNA (Sarngadharan et al., 1972), a primary etiological factor of the mammary gland neoplasia in mice. Despite these independent findings, no actual candidate virus for human cancer was defined at that time. Years later, when Ono and colleagues (1986) (Ono et al., 1986) described and sequenced a complete endogenous retroviral sequence, later named human MMTV-like virus (HML-1-10) (Franklin et al., 1988) from superfamily HERV-K, the studies attempted to relate this retroelement with cancer began.

In an independent line of investigation, electron microscopy analysis of teratocarcinoma cell lines (Tera-1) revealed retroviral-like particles budding from the cells, and named it as “*human teratocarcinoma derived virus* (HTDV)”. The researchers however could not isolate the viruses using fresh cells, suggesting that the particles were non-infectious (Bronson et al., 1979; Löwer et al., 1984). Years later, it was finally demonstrated through Western-blot with anti-HERV-Kgag antibodies that the HTDV was precisely the just described HERV-K (Boller et al., 1993).

The studies on HERV-K and cancer then intensified and many other types of cancer were also associated with the K family, as melanoma, leukemia, prostate, colorectal, brain, etc (**Figure 1**) (Brodsky et al., 1993; Sauter et al., 1995; Büscher et al., 2005; Manca et al., 2022). The studies spanned from the mRNA measures and the utility of HERV-K transcripts quantification as biomarkers for progression (Contreras-Galindo et al., 2008), to the antibody anti-HERVs detection and mechanisms of malignancy and potential interventions (Li et al., 2022; Manca et al., 2022; Zanrè et al., 2024). Although HERV-K is still the most explored HERV in cancer studies (**Figure 1**), other families were also implicated in tumorigenesis as well. Colorectal cancers (CRCs) rank among the most prevalent cancers globally, characterized by notably low 5-year survival rates of less than 70% in many American and European countries. Over the past decade, research attention has increasingly focused on the impact of HERV elements on CRCs, particularly involving HERV-H. In 2015, researchers detected a significant elevation of HERV-H gag, pol, and env RNA levels in CRC patients, with HERV-H loci on chromosomes Xp22.3 and 20p11.23 being the most active (Pérot et al., 2015). The same study identified a correlation between HERV-H expression levels and lymphnode invasion. A more recent study aimed to investigate the HERV-H LTR-association protein 2 (HHLA2) in colorectal cancer and its association with clinicopathological features. The authors found that HHLA2 expression was significantly upregulated in CRC tissues compared to adjacent and normal tissues. The expression level was strongly correlated with deeper tumor invasion, lymphnode metastasis, advanced clinical stage, and poorer survival. Critically, a significant inhibition of tumor proliferation, migration, and invasion observed upon silencing HHLA2 in CRC cells pointed the HERV-H as a promising target for drug development in CRC treatment (Wang H. et al., 2024).

Based on the compilation of studies and data accessible through CancerHERVdb (Stricker et al., 2023a), it is evident that HERV expression is not random but exhibits a highly coordinated pattern, where specific families or loci are implicated in particular types of cancer, but not in others. This demonstrates that the expression is likely not just a consequence of epigenetic changes after cellular transformation, but rather, the HERV regulation may be intricately linked to malignancy and/or proliferation. A retrotranscriptome analysis of head and neck cancer and adjacent normal tissue revealed 1078 HERVs from different families exhibited distinct expression patterns between tumor and healthy tissue. While most of them belong to HERV-H family and were overexpressed only in tumor tissues (Kolbe et al., 2020), HERV-K, the most active family, was not related to this type of cancer in this study.

Mechanisms. While the precise roles of HERVs in carcinogenesis remain inconclusive, a range of diverse functions have been proposed. These include acting as noncoding RNAs, signaling proteins, and transcriptional regulators. Endogenous Betaretrovirus as HERV-K, or Spumaretrovirus as HERV-L code additional spliced genes such as rec and np9 (HERV-K), or tas/bel1 and bet (HERV-L) (Stricker et al., 2023b). Particularly, the two HERV-K derived proteins have been extensively investigated for their potential involvement in tumorigenesis. Both are known to interact with the promyelocytic leukemia zinc finger protein (PLZF) tumor suppressor, disrupting the transcriptional repression of the c-myc proto-oncogene by PLZF, thereby stimulating cell proliferation (Denne et al., 2007). Np9 has also been found to disrupt the MDM2 ubiquitin ligase activity towards p53 within the cell nucleus, leading to an increase in p53 levels (Heyne et al., 2015).

Other mechanisms include the participation of LTR or their regulatory elements driving the expression of nearby oncogenes. A comprehensive genome-wide analysis revealed an enrichment of binding sites for transcription factors such as CTCF, TP53, Sox2, and ESR1 within different HERV LTRs (Bourque et al., 2008). Wang and colleagues also described that HERV-LTRs containing p53 binding site are capable to promote the activation of downstream genes associated with p53 (Wang et al., 2007). Based on an aberrant activation expression of HERV-K among different breast cancer samples, Liang et al. (2024) (Liu et al., 2023) analyzed 91 HERV-K loci and nearby genes, investigating their impact on the tumor microenvironment (TME). Among the host genes close to the HERVs, some emerged as key genes associated with poor breast cancer prognosis. These genes were functionally enriched in immune-related pathways, impacting breast cancer development potentially by modulating TME immune cell infiltration.

## 5 HERVs in neuroinflammatory disorders – a brief overview

Among neuroinflammatory disorders potentially linked to HERVs, Multiple Sclerosis (MS) has been the subject of extensive research over the past decades. As previously reported by our group in a comprehensively chronological evidence-based review (Rangel et al., 2022), several evidence contributed to strengthening the etiological role of HERVs on MS pathogenesis. Not only do MS individuals present a higher level of HERV expression than healthy individuals (Perron et al., 1997; Olival et al., 2013; Horssen et al., 2016), as well as HERV-W env protein was already detected in active white matter lesion of MS (Antony et al., 2004; Perron et al., 2005). More recently, it was described that in addition to HERV-W, various other HERV families are also upregulated in MS individuals (Nali et al., 2022). Despite of it, the putative role of HERVs in MS pathogenesis was only demonstrated with the W family. Perron and colleagues first described that HERV-W env protein was able to induce MS in humanized mice (Perron et al., 2013) and, more recently, it was demonstrated the ability of HERV-W env protein to interfere in the deterioration of glial cells, which could potentially impact the glial repair (Gruchot et al., 2023b).

In addition to MS, HERVs were also associated with other neuroinflammatory disorders, as Alzheimer’s Disease (AD) and Amyotrophic lateral sclerosis (ALS), both discussed below in this review, Parkinson’s Disease (PD), Bipolar Disorder (BD), Schizophrenia (SZ), and Myalgic encephalomyelitis/Chronic Fatigue Syndrome (ME/CFS) are also field for HERV investigation (see **Figure 1**). While distinct pathological mechanisms characterize each of these conditions, they all involve a neuroinflammatory component (Goldsmith et al., 2016; Tansey et al., 2022). Regarding PD, although limited information exists on the dynamics of HERV expression throughout its pathogenesis, previous studies have reported a high prevalence of unfixed HERV-K insertions in the genomes of PD patients, highlighting the polymorphic nature of HERV-K distribution in this disease (Wildschutte et al., 2016; Wallace et al., 2018).

Increased levels of HERV-K, HERV-W, and HERV-H expression have been observed in BD and SZ, along with an association between higher concentrations of pro-inflammatory cytokines and HERV-W antigenemia, suggesting a potential inflammatory modulation mediated by HERVs (Karlsson et al., 2001; Perron et al., 2012; Tamouza et al., 2021). Notably, a transcriptional profile of HERV-W, LTR17, HERV-H, and HERV-K10 has been identified in the post-mortem brains of SZ and BD patients (Frank et al., 2005), and among all families, the HML-2 (HERV-K10) was the only consistently overrepresented in both groups. Differently than MS or other better-studied diseases, the relation of HERVs and ME/CFS has been far less explored. Our group investigated HERV-K and HERV-W expression in moderate and severe ME/CFS cases and described increased HERV-K expression only in individuals with moderate CFS (Rodrigues et al., 2019). Using immunological approaches, a study reported the presence of HERV-K gag, HERV-K18 env, HERV-FRD, and HERV-R proteins in duodenal biopsies of CFS patients (De Meirleir et al., 2013), suggesting a potential differentiation of immunoreactive cell phenotypes and presenting an unique scenario involving broad anti-HERV immunoreactivity in plasmacytoid dendritic cells. Finally, a comprehensive study comparing retroelements activity between Fibromyalgia and Chronic Fatigue, two distinct diseases but with confounding initial symptoms, mapped retroelements differentially expressed in both diseases. They found that particular HERVs exhibited either upregulation or downregulation in each group, with significant patterns observed within the ME/CFS cohort. These differences correlated with variations in immune gene expression and patient symptomatology, endorsing the subtyping of ME/CFS patients and confirming the presence of immunological disturbances in this condition (Giménez-Orenga et al., 2023).

## 6 Neurodegenerative diseases and human endogenous retroviruses

### 6.1 Amyotrophic lateral sclerosis

Also known as motor neuron disease, is a rare and fatal neurodegenerative condition characterized by the loss of motor neurons in the brain and spinal cord. Its incidence is slightly higher in populations of predominantly European descent, with approximately 2 cases per 100,000 person-years, with a typical survival ranging from 3 to 5 years after diagnosis. The incidence of ALS rises with age, being highest among individuals aged 60 to 79 years (Feldman et al., 2022). ALS manifests in distinct phenotypes, being bulbar onset and spinal onset (cervical, lumbar) the most prevalent presentations, accounting for approximately a quarter to a third of cases respectively. Many genetic variants associated with ALS have been identified, impacting individuals both with and without a family history of the disease (Feldman et al., 2022). Despite significant progress, the etiology of many sporadic ALS cases remains uncertain.

It has been almost 15 years since the first evidence of the involvement of HERVs in ALS pathogenesis (Douville et al., 2011). Since then, accumulated findings have included elevated (up to 3-fold increase) expression of retroviral genes in the brains of ALS patients; the presence of HERV-K-env protein in the cerebrospinal fluid (CSF), and neurons; HERV-K env protein in neuronal extracellular vesicles (NEV) of patients, with higher concentrations in patients with worse clinical conditions; higher levels of antibody concentration against HERV-K in CSF and serum of ALS patients, and more (Douville et al., 2011; Li et al., 2015; Arru et al., 2018; Li et al., 2022; Steiner et al., 2022). Yet, the implications of HERV expression for ALS pathogenesis are not fully understood.

Some hypotheses consider the ability of HERV-K-env to stimulate immune responses, resulting in increased production of pro-inflammatory cytokines, including IFN-γ, MIP-1α, and TNF-α (Arru et al., 2021). HERV-K has been shown to induce protein aggregates and neurotoxicity in animal models, causing significant changes in neuronal morphology *in vivo* (Li et al., 2022). Apart from neurotoxicity, a concealed HERV-K encoded protein within the env gene is expressed during an inflammatory response, impacting inflammation pathways (Di Curzio et al., 2020).

Considering the putative role of HERV expression in ALS development or pathogenesis, the effect of combined antiretroviral therapy with abacavir, lamivudine, and dolutegravir on the HERV-K (HML-2) transcription levels was investigated in ALS patients. After 6 months of treatment, a significant proportion of participants (82%), exhibited a reduction in HML-2 load compared to pre-treatment levels. Notably, differences in the evolution of certain clinical outcomes were also observed (Gold et al., 2019; Garcia-Montojo et al., 2021). This pivotal study not only presents a potential benefit of antiretroviral therapy as a possible limiter of ALS progression but also corroborates the involvement of HERVs from the K family in ALS.

### 6.2 Alzheimer’s disease

The most common age-related neurodegenerative disorder and is characterized by progressive memory loss and cognitive dysfunction. AD induces the loss of motor functions and personality alterations, ultimately leading the patient to death (Mattson, 2004). Histopathologically, AD is marked by extracellular senile plaques (SPs) resulting from the aggregation of Αβ Amyloid protein, and by intracellular neurofibrillary tangles (NFTs). Based on the amyloid hypothesis, proposed in 1992, Aβ is considered the key factor that triggers the onset and progression of Alzheimer’s Disease (Hardy and Higgins, 1992). However, accumulating evidence from genetic, imaging, and biochemical data suggests that Aβ is only part of the disease, indicating a much more complex etiology. Tau deposition precedes grey matter atrophy, indicating that misfolded Tau may be a major driver of AD pathogenesis (Vojtechova et al., 2022).

As it is extensively described for Multiple Sclerosis, some HERV families are also overexpressed, and several active loci have been identified in Alzheimer’s Disease (Nali et al., 2022). Notably, the most active HERVs are often located near immune response genes, suggesting a potential (dis) regulation of the immune system by these retroviruses (Dawson et al., 2023). By assuming that HERV-K RNA is capable of inducing CNS (central nervous system) injury, Dembny et al. (2020) (Dembny et al., 2020),demonstrated that the silencing of HERV-K RNA protected neurons from the neurotoxicity in animal models. The HERV-K inhibition also prevented neurodegeneration and microglial activation (Dembny et al., 2020). In the same study, the authors detected HERV-K transcripts in almost 90% of the cerebrospinal fluid (CSF) of the individuals with Alzheimer’s Disease against only 2% of the control subjects. Upon sequencing the transcripts, they identified the enrichment of LTR5_Hs/HERV-K exclusively in AD patients, providing additional evidence for an association between a very specific HERV-K activation and AD.

### 6.3 Prion diseases

As discussed above, the misfolding of proteins, such as the microtubule-binding protein Tau, is linked to highly prevalent neurodegenerative diseases, notably Alzheimer’s disease. While mutations in aggregation-prone proteins explain some cases of familial neurodegenerative diseases, the etiologies of spontaneous diseases, including prion diseases, remain unknown. Prion diseases can be etiologically categorized as sporadic, genetic, or acquired through the infection of prion-contaminated agents (Prion Biology and Diseases, 2024). The majority of human prion diseases fall under the classification of Creutzfeldt-Jakob disease (CJD), in its sporadic form, while 10-15% are attributed to mutations in the prion protein gene (PRNP). Consistent with other prion diseases, CJD is characterized by the accumulation of the alternative folded PRNP, amyloid plaques, spongiform vacuolation, astrocytic proliferation, leading to neuronal cell loss (Prion Biology and Diseases, 2024).

There is a body of studies covering the ERVs dysregulation in prion diseases. The first evidence dates back to 1999 when researchers showed in murine models a relation between the scrapie infectious process and MuLV replication (Lachmann et al., 1999). In the same line, it was later demonstrated that infection of a senescence-accelerated mouse strain (SAMP8) that develops active ecotropic MuLV with scrapie led to an increase in the MuLV titer (Jeong et al., 2002). These observations were corroborated by the presence of vacuoles within the cytoplasm of MuLV-positive neurons, and the extracellular space surrounding these neurons exhibited lytic alterations. Later, in non-human primates, it was detected a dysregulation of gamma and beta-like ERVs in response to BSE (bovine encephalopathy spongiform) agent on both the RNA and the protein level (Greenwood et al., 2011).

In humans, it was demonstrated that HERV-W, HERV-L, FRD, and ERV-9 transcripts exhibited a significant increase in the cerebrospinal fluid (CSF) of individuals with sporadic Creutzfeldt-Jakob disease compared to normal control CSF. Moreover, when compared to individuals with other neurodegenerative diseases manifesting similar symptoms to CJD, such as dementia, the incidence rates of HERV-W and HERV-L were notably higher in the CSF of sporadic CJD patients (Jeong et al., 2010).

Although a bit controversial, it has also been proposed that the intercellular trafficking of prions may be partially facilitated by hitchhiking on endogenous retroviral particles (Fevrier et al., 2004). This hypothesis comes from observations that PrP interacts with retroviral RNA and with nucleoprotein structures of HIV, which include Gag protein (Gabus et al., 2001b; Gabus et al., 2001a). Additionally, neuroblastoma cells infected with scrapie and Creutzfeldt-Jakob disease agents produce intracellular 25-nm virus-like particles, that together with exosomal particles (Fevrier et al., 2004), would help in the spreading of misfolded proteins (Gabus et al., 2001b; Gabus et al., 2001a; Greenwood et al., 2011).

## 7 HERV in rheumatic diseases

Rheumatic diseases are a group of disorders that may also be associated with aging, or at least, with the epigenetic dysregulation of the normal aging process.

### 7.1 Fibromyalgia

A complex disease with an unknown etiology characterized by widespread pain throughout the body and increased pain sensitivity, significantly compromising individuals’ quality of life (Wolfe et al., 2011). Patients with FM exhibit overlapping symptoms with those with Myalgic Encephalomyelitis/Chronic Fatigue Syndrome (ME/CFS), though they are now recognized as distinct conditions. However, inflammatory dysregulation is present in both diseases (Strawbridge et al., 2019; Yao et al., 2023). As far as we know, only a few studies were performed to the understanding of the HERVs putative involvement in FM and in ME/CSF. One study described increased levels of HERV-H, K, and W in patients with FM compared to healthy controls. Interestingly, they also reported a positive correlation between HERV expression and pro-inflammatory cytokines (Ovejero et al., 2020), suggesting that HERVs may play a role in the inflammatory response commonly associated with FM patients. As already mentioned in this review, a more recent study (Giménez-Orenga et al., 2023) deeply investigated the HERV expression profile in FM and ME/CSF, and described a family-specific HERV deregulation in the immune cells of individuals with ME/CFS and FM, with a heightened HERV dysregulation in the ME/CFS (66 families) compared to FM (22 families), supporting the biological distinctions between ME/CFS and FM. Fibromyalgia may also occur with other chronic pain conditions like Osteoarthritis (OA) and Rheumatoid Arthritis (RA).

### 7.2 Osteoarthritis

The most common chronic articular-associated disease, affecting small, medium, and large joints. The knee is most frequently affected in up to 10% of men and 13% of women aged above 60 years (Jang et al., 2021). Despite being one of the oldest documented diseases, its etiology remains unknown. While the expression of endogenous retroviruses has been well covered in RA (Rangel et al., 2022), few data on OA have been published in this matter. In a search for exogenous (herpesviruses mostly) and endogenous retroviruses in cartilage and chondrocytes from osteoarthritis patients, transcripts of endogenous retroviruses, specifically HERV-WE1 and WE2, were detected in 15 of 17 patients, while no exogenous viruses were found. More interestingly, retroviral-like particles were observed in chondrocyte cultures (Bendiksen et al., 2014). In parallel, researchers also investigated the expression of ERV-3, a single copy provirus from the HERV-R family, in patients with OA and RA and found transcripts only in OA individuals (Nelson et al., 2010). They suggested that this retroelement could serve as a biomarker for OA, given that the expression of this ERV is not commonly detected, but the real impact of its activity in these patients would require further studies.

## 8 Exogenous viruses and HERVs

Extreme inflammatory manifestations and neurological manifestations such as encephalopathies, dementias, Guillain-Barret syndrome, and degenerative syndromes can occur as a consequence of infection by exogenous viruses, such as Flaviviruses, retroviruses such as the Human Immunodeficiency virus (HIV), and human T-cell lymphotropic virus (HTLV), and coronaviruses as Severe acute respiratory syndrome coronavirus 2 (SARS-CoV-2). Interestingly, there is extensive literature showing that these viruses interact with endogenous retroviruses, increasing their transcription. Some studies even highlight that this activity can be linked to the progression of systemic diseases. Unfortunately, few of these studies emphasize the activity of HERVs in the neurological manifestations caused by these viruses, which would be a topic of utmost relevance.

### 8.1 HIV-1

Several studies have explored the potential interaction between HIV and HERV, particularly HERV-K(HML2), demonstrating increased HERV activity in the presence of HIV-1 or only its proteins. The majority of the studies focused on detecting HERV transactivation events, and on understanding how the immune response is affected. Contreras-Galindo repeatedly described HERV-K (HML-2) viral RNA in the plasma of HIV-1-infected patients, occasionally at astonishing high titers (up to 10E10 RNA copies/ml) (Contreras-Galindo et al., 2006). He also illustrated that HERV-K expression is higher in patients with non-suppressive anti-retroviral therapy (ART) than those with a suppressive regimen and that increased HERV-K RNA titers often preceded HIV-1 rebounds. Based on these findings, the authors proposed that HERV-K load could be a useful predictor of HIV-1 reactivation (Contreras-Galindo et al., 2007a). *In vitro* experiments also appear to confirm it.

Microarray analysis of HIV-1 infected cell cultures described upregulation for HERV-T, ERV-9, and HERV-E, as well as HERV-K (Vincendeau et al., 2015). In the same experiment, the authors showed that cells infected *de novo* by HIV-1 showed a stronger signal of the HERV-K (HML-2) group than persistently infected cells. In an experiment to assess whether the HIV-1 subtype could differentially influence the transactivation of HERVs, Li, and co-workers found an increase in the transcriptional levels in the HERV-K gag region in HIV-1 B subtype-infected patients, whereas the transcriptional levels of the HERV-K pol region were increased in CRF01_AE and CRF07_BC recombinant-infected patients (Li et al., 2021).

The transactivation of HERVs by HIV is not entirely clear, but a better-explored mechanism would involve HIV- Tat protein. Gonzalez-Hernandez et al. identified Tat as a putative transactivator of HERV-K, specifically the HML-2 subtype, through its interaction with cellular transcription factors NF-κB and NF-AT (Gonzalez-Hernandez et al., 2012). This activity caused different impacts on the proviruses, as evidenced in transcriptome analyses where 26 proviruses showed significant expression, but 12 were silenced, indicating a complex interaction between Tat and HML-2 (Gonzalez-Hernandez et al., 2014). Contreras-Galindo et al. proposed a mechanism in which Tat would assist HERV-K activation. They reported that some proviruses, especially K111 (HML-2), located in a centromeric region, are expressed in the presence of Tat (Contreras-Galindo et al., 2013). The role of Tat seemed to be critical since this protein drives a transition from heterochromatin to euchromatin by activating histone acetylases (Easley et al., 2010).

However, there is not unanimous agreement across the studies regarding the increased HERV activity driven by HIV-1. Karamitros and colleagues systematically tested 236 plasma of HIV-infected individuals and reported all of them to be negative for HERV-K expression. The authors developed a rigorous protocol to eliminate any possible contamination with genomic DNA (gDNA) and therefore, believe that the discrepancy in their results concerning studies that find HERV-K transcripts is primarily due to this reason (Karamitros et al., 2016).

Regardless, HERV proteins appear to be capable of activating the host immune responses. It was suggested that some HERVs may play a crucial role in the cellular and humoral immune response to HIV-1 infection. Contreras-Galindo et al. (2007) observed HERV-K Gag protein expression in HIV-1 infected T-cells *in vivo* and suggested a potential implication in disease development. But in parallel, Garrison demonstrated that HIV-1 seropositive patients also exhibited a T-cell response to a HERV antigen (HERV-L IQ10 peptide) that shares similar regions to HIV epitopes. The authors proposed a mechanism wherein HIV-1 infected cells expressing HERVs would elicit a stronger immune response against them. Critically, the level of T-cell responses to HERV was inversely correlated to HIV-1 plasma viral load in all individuals included in the study (Garrison et al., 2007).

In addition to the cellular response, HIV infection also induces a humoral response through HERV activity. Michaud et al. (2014) demonstrated that HERV-K Env mRNA is expressed in the surface and transmembrane regions of cells infected by HIV-1, and antibodies against HERV-K Env protein are present in such patients. They also reported that elite controllers had a higher titer of anti-HERV-K compared to non-elite controllers ​​ (Michaud et al., 2014).

People living with HIV (PLWH) may develop a spectrum of cognitive, motor, and mood alterations known as HIV-associated neurocognitive disorder (HAND). HAND encompasses a range of neurocognitive impairments, including asymptomatic neurocognitive impairment (ANI), mild neurocognitive disorder (MND), and HIV-associated dementia (HAD). HAND affects over 50% of PLWH, and the risk of developing such disorders is heightened with age. HIV doesn’t directly infect neurons, instead, the CNS is a viral reservoir since the virus resides in different cells within the nervous system, including macrophages, microglia, and astrocytes. In the long term, it is implicated in neuronal injury through neurotoxic viral factors, triggering processes of neuroinflammation and neurodegeneration (Wahl and Al-Harthi, 2023).

A temporal pattern can be observed in the activation of HIV and HERV-K in the brains of PLWH. Notably, there is an increase in HERV-K activation preceding the manifestation of clinical symptoms of neurocognitive impairment (Douville et al., 2011). Conversely, heightened HERV-K env expression in cortical neurons of HIV-infected individuals has been associated with the inhibition of HIV replication in these cells and neuron protection (Bhat et al., 2014). However, over the long term, neuronal HERV-K expression results in neurite retraction and neuronal death, aligning with the observed outcomes in HIV-associated neurocognitive disorders (Dembny et al., 2020).

### 8.2 HTLV

Human T-lymphotropic virus (HTLV) is the causative agent of chronic progressive myelopathy (TSP/HAM) in which lesions of the central nervous system (CNS) are associated with infiltration of HTLV-1-infected T-cells and to the adult T-cell leukemia (ATL). It was the first documented human retrovirus, but, unlike HIV, much remains to be understood about the intricate presentations and syndromes caused by this virus (Poiesz et al., 1980; Bangham et al., 2015).

The studies investigating the potential transactivation of HERV by HTLV were possibly inspired by the fact that HTLV causes neurological syndromes with an extremely inflammatory profile, involving astrocytes and presenting similarities to other neurodegenerative diseases like MS and ALS, in which the link with HERVs is recognized (Tanajura et al., 2015). Additionally, the growing literature on HERV transactivation by other retroviruses as HIV-1 possibly contributed to the interest in the HERV/HTLV putative link. However, the literature on HERV and HTLV remains quite scarce and somewhat controversial.

A study by Toufaily and collaborators showed that the HTLV Tax protein can activate HERV LTR. In their work, the authors transfected HTLV-LTR into Jurkat cells culture expressing Tax, and an increase in HERV-W and HERV-H LTR activity was observed. Experimental evidence indicated that this activation is carried through the transcription factor CREB (Toufaily et al., 2011). Perzova. in 2013, described anti-HERV-K10 antibodies in patients with HTLV-associated myelopathy. Of 16 patients with myelopathy, 14 had anti-HERV-K Gag antibodies and 15 had anti-HERV-K10 Pol antibodies (Perzova et al., 2013).

Conversely, based on the abovementioned studies, PBMC from 15 HTLV-1-infected subjects were screened for T-cell responses against HERV-K(HML-2) Gag and Env and also against other HERV families. The study however failed to demonstrate cellular responses against HERVs in HTLV individuals (Jones et al., 2013).

### 8.3 SARS-COV-2

Following the line of interactions between HERVs and exogenous viruses with an inflammatory profile of pathogenesis, studies on HERVs and SARS-CoV-2 have been rapidly emerging. In addition to the systemic inflammation caused by this virus, a range of neurological manifestations (long COVID or neuro-COVID) have been and are still being described. Some studies on neurocovid relate the symptoms of both acute and non-acute manifestations as a putative consequence of a persistent inflammatory state in the central nervous system. This is because the virus is rarely found in cerebrospinal fluid samples, suggesting that tissue damage is indirect, via the stimulation of inflammatory factors and other involved genes (Matos A de et al., 2021; Etter et al., 2022).

Several studies have assessed the HERVs profile in COVID-19. A transcriptome in SARS-CoV-2-infected patients revealed a distinct distribution of HERVs transcripts based on exposure to SARS-CoV-2, regardless of the severity of COVID-19, where many HERV families are differentially expressed compared to healthy individuals (Marston et al., 2021). Another group investigated the impact of SARS-CoV on the HERVs transcriptome using publicly available transcriptome data from cells infected by SARS-CoV-2 and found overexpression of HERV-H, HERV-W, HERV-3, HERV-K, and HERV-E families. The expression profile however was distinct in cell lines compared to clinical samples, where syncytin-1 and syncytin-2 transcripts were markedly increased in clinical samples. In this same work, analysis of ChIP-Seq data showed that TEs differentially expressed in SARS-CoV-2 infection were enriched for binding sites for transcription factors involved in immune responses.

The results of this study are in line with the previous one published by Balestrieri et. al (2021).,, where an increase in HERV-W was observed in hospitalized COVID-19 patients. The authors highlighted that the expression of HERV-W envelope in lymphocytes at the time of sampling reflected the respiratory outcomes throughout the hospitalization, suggesting its involvement in the pathogenesis of the disease. Also, the percentage of CD4+ cells positive for HERV-W ENV proved to be a more specific marker for predicting the need for respiratory support compared to IL6 concentration in plasma (Balestrieri et al., 2021).

A comprehensive report including patients infected with different SARS-CoV-2 variants investigated different sites using a range of methods. Immunohistology analyses found that HERV-W ENV is expressed in postmortem tissues of lungs, gut, heart, brain parenchyma, and nasal mucosa from acute COVID-19 patients and the sites where HERV-W were found correlated to the clinical manifestations observed in the donor patients (Charvet et al., 2023). Collectively, these findings indicate that HERV-W ENV serves not only as a COVID-19 severity biomarker but it may be functioning as an additional pathogenic factor influencing the severity of the disease.

### 8.4 Epstein Barr virus

The Epstein-Barr virus, a member of the herpesviridae family (herpesvirus type IV), is the causative agent of infectious mononucleosis, commonly known as the “kissing disease” due to its frequent transmission through saliva. This virus is globally prevalent, infecting around 95% of the adult population. EBV also causes a latent infection in immortalized B cells and, depending on the individual’s immunological condition, it can be reactivated and induce other more serious conditions. The individual’s immune system plays a crucial role during the course of infection (Yu and Robertson, 2023).

The immune mechanisms involving EBV infection and HERVs activation have been studied for decades. In 2001 Sutkowski and colleagues found that EBV induces a superantigen (SAg) activity by transactivating HERV-K18 env in infected B cells, leading to a T cell response (Sutkowski et al., 2001). Later, the same group showed that the latent membrane protein 2A (LMP-2A) present in the EBV virion was sufficient to trigger this immune response (Sutkowski et al., 2004). It is hypothesized that EBV, by causing a latent infection, triggers a superantigen (SAg) activity that prompts memory-infected B cells to become immortalized. However, in 2006, Hsiao et al. outlined an alternative pathway wherein EBV induces SAg activity through viral latent proteins LMP-2A, LMP-1, and its cellular receptor, CD21. This suggests that this transactivation may serve additional functions beyond inducing cell immortality during the latent phase, potentially involving processes related to viral entry into cells (Hsiao et al., 2006). In 2009, Hsiao elucidated the pathway in which EBV infection induced the transactivation of HERV-K18 env, showing that an immunoreceptor tyrosine-based activation motif (ITAM) receptor is important for this activity. In addition, they showed that elements essential to this transactivation are located downstream the HERV-K18 env gene (Hsiao et al., 2009).

HERV-K Gag is also activated in EBV-triggered immortalized cells from patients with multiple sclerosis (MS) (Wieland et al., 2022). Regarding HERV-W, which is strongly associated with MS pathogenesis, there is evidence of HERV-W gp350 transactivation by EBV infection in peripheral blood mononuclear cells (PBMC) from MS patients (Mameli et al., 2012). A proposed mechanism suggests that EBV and HERV-W may cooperate in the pathogenesis of MS. Mameli (Mameli et al., 2012) suggests that systemic activation of HERV-Wenv can lead to immunopathogenic events due to its super antigenic properties. This, in turn, may result in toxicity against oligodendrocytes in the brain, leading to inflammation, demyelination, and axonal damage. Therefore, the immunological inability to mitigate the expression and transactivation between EBV and HERV-W would impact on the MS immunopathology.

## 9 Concluding remarks

The expression of HERVs in the host’s genetic regulation has been proven to be fundamental for the homeostasis and balance of various functions, both early in life and during the processes of aging and senescence. Conversely, the involvement of endogenous retroviruses in degenerative, rheumatic, and other diseases presenting a range of clinical manifestations is also recurrently demonstrated, although definitive conclusions regarding the impact of their expression on such diseases have not been demonstrated. Yet, the expression of these elements does not seem to be merely a consequence of epigenetic dysregulation or a secondary stimulus from cellular and pro-inflammatory factors. Instead, it appears to be an orchestrated selection of elements that are up or down-regulated, in very specific situations. Entire HERV families can be upregulated in one specific disease, viral particles are formed, and anti-HERV antibodies are present, whilst the same families are down-regulated in another. When required, only specific loci from a given HERV family are switched on or off dictating the fate of the cells or tissues in which this process is taking place. To what extent does HERV contribute to the host genetic network? How can we use this information to control HERV-related diseases? These questions still lack definitive answers. However, the prospects seem promising.

## Author contributions

AS: Writing – original draft, Data curation, Investigation. BG: Data curation, Investigation, Writing – original draft. SS: Investigation, Writing – original draft. GC: Writing – original draft, Data curation. AN: Writing – original draft. LN: Writing – original draft, Formal analysis, Supervision. AB: Supervision, Writing – original draft, Conceptualization. CR: Conceptualization, Supervision, Writing – original draft, Funding acquisition, Writing – review & editing.

## Supplementary material

Supplementary File 1 Table containing all entries (with respective D.O.I and links) used to construct the **Figure 1**.

## References

[B1] Aftab A. Shah A. A. Hashmi A. M. (2016). Pathophysiological role of HERV-W in schizophrenia. J. Neuropsychiatry Clin. Neurosci. 28, 17–25. doi: 10.1176/appi.neuropsych.15030059 26404170

[B2] Antony J. M. van Marle G. Opii W. Butterfield D. A. Mallet F. Yong V. W.. (2004). Human endogenous retrovirus glycoprotein–mediated induction of redox reactants causes oligodendrocyte death and demyelination. Nat. Neurosci. 7, 1088–1095. doi: 10.1038/nn1319 15452578

[B3] Arru G. Galleri G. Deiana G. A. Zarbo I. R. Sechi E. Bo M.. (2021). HERV-K modulates the immune response in ALS patients. Microorganisms. 9, 1784. doi: 10.3390/microorganisms9081784 34442863 PMC8399181

[B4] Arru G. Mameli G. Deiana G. A. Rassu A. L. Piredda R. Sechi E.. (2018). Humoral immunity response to human endogenous retroviruses K/W differentiates between amyotrophic lateral sclerosis and other neurological diseases. Eur. J. Neurol. 25, 1076–1e84. doi: 10.1111/ene.13648 29603839

[B5] Babu M. M. Luscombe N. M. Aravind L. Gerstein M. Teichmann S. A. (2004). Structure and evolution of transcriptional regulatory networks. Curr. Opin. Struct. Biol. 14, 283–291. doi: 10.1016/j.sbi.2004.05.004 15193307

[B6] Balestrieri E. Minutolo A. Petrone V. Fanelli M. Iannetta M. Malagnino V.. (2021). Evidence of the pathogenic HERV-W envelope expression in T lymphocytes in association with the respiratory outcome of COVID-19 patients. eBioMedicine. 66, 103341. doi: 10.1016/j.ebiom.2021.103341 33867312 PMC8082064

[B7] Balestrieri E. Pica F. Matteucci C. Zenobi R. Sorrentino R. Argaw-Denboba A.. (2015). Transcriptional activity of human endogenous retroviruses in human peripheral blood mononuclear cells. BioMed. Res. Int. 2015, e164529. doi: 10.1155/2015/164529 PMC4334862 25734056

[B8] Bangham C. R. M. Araujo A. Yamano Y. Taylor G. P. (2015). HTLV-1-associated myelopathy/tropical spastic paraparesis. Nat. Rev. Dis. Primer. 1, 1–17. doi: 10.1038/nrdp.2015.12 27188208

[B9] Bannert N. Kurth R. (2006). The evolutionary dynamics of human endogenous retroviral families. Annu. Rev. Genomics Hum. Genet. 7, 149–173. doi: 10.1146/annurev.genom.7.080505.115700 16722807

[B10] Bektas A. Schurman S. H. Sen R. Ferrucci L. (2017). Human T cell immunosenescence and inflammation in aging. J. Leukoc. Biol. 102, 977–988. doi: 10.1189/jlb.3RI0716-335R 28733462 PMC5597513

[B11] Belshaw R. Dawson A. L. A. Woolven-Allen J. Redding J. Burt A. Tristem M. (2005). Genomewide screening reveals high levels of insertional polymorphism in the human endogenous retrovirus family HERV-K(HML2): implications for present-day activity. J. Virol. 79, 12507–12514. doi: 10.1128/JVI.79.19.12507-12514.2005 16160178 PMC1211540

[B12] Bendiksen S. Martinez-Zubiavrra I. Tümmler C. Knutsen G. Elvenes J. Olsen E.. (2014). Human endogenous retrovirus W activity in cartilage of osteoarthritis patients. BioMed. Res. Int. 2014, e698609. doi: 10.1155/2014/698609 PMC4130134 25136615

[B13] Bergallo M. Fava P. Galliano I. Novelli M. Montanari P. Daprà V.. (2018). Molecular genetic analyses of human endogenous retroviral elements belonging to the HERV-P and HERV-R family in primary cutaneous T-cell lymphomas. J. Eur. Acad. Dermatol. Venereol. 32, e297–e298. doi: 10.1111/jdv.14840 29405482

[B14] Bhat R. K. Rudnick W. Antony J. M. Maingat F. Ellestad K. K. Wheatley B. M.. (2014). Human endogenous retrovirus-K(II) envelope induction protects neurons during HIV/AIDS. PloS One 9, e97984. doi: 10.1371/journal.pone.0097984 24988390 PMC4079299

[B15] Boller K. König H. Sauter M. Mueller-Lantzsch N. Löwer R. Löwer J.. (1993). Evidence that HERV-K is the endogenous retrovirus sequence that codes for the human teratocarcinoma-derived retrovirus HTDV. Virology. 196, 349–353. doi: 10.1006/viro.1993.1487 8356806

[B16] Bourque G. Leong B. Vega V. B. Chen X. Lee Y. L. Srinivasan K. G.. (2008). Evolution of the mammalian transcription factor binding repertoire via transposable elements. Genome Res. 18, 1752–1762. doi: 10.1101/gr.080663.108 18682548 PMC2577865

[B17] Brodsky I. Foley B. Haines D. Johnston J. Cuddy K. Gillespie D. (1993). Expression of HERV-K proviruses in human leukocytes. Blood. 81, 2369–2374. doi: 10.1182/blood.V81.9.2369.2369 7683217

[B18] Bronson D. L. Fraley E. E. Fogh J. Kalter S. S. (1979). Induction of retrovirus particles in human testicular tumor (Tera-1) cell cultures: an electron microscopic study. J. Natl. Cancer Inst. 63, 337–339. doi: 10.1093/jnci/63.2.337 287828

[B19] Büscher K. Trefzer U. Hofmann M. Sterry W. Kurth R. Denner J. (2005). Expression of human endogenous retrovirus K in melanomas and melanoma cell lines. Cancer Res. 65, 4172–4180. doi: 10.1158/0008-5472.CAN-04-2983 15899808

[B20] Cardelli M. (2018). The epigenetic alterations of endogenous retroelements in aging. Mech. Ageing Dev. 174, 30–46. doi: 10.1016/j.mad.2018.02.002 29458070

[B21] Carter T. A. Singh M. Dumbović G. Chobirko J. D. Rinn J. L. Feschotte C. (2022). Mosaic cis-regulatory evolution drives transcriptional partitioning of HERVH endogenous retrovirus in the human embryo. eLife 11, e76257. doi: 10.7554/eLife.76257.sa2 35179489 PMC8912925

[B22] Charvet B. Brunel J. Pierquin J. Iampietro M. Decimo D. Queruel N.. (2023). SARS-CoV-2 awakens ancient retroviral genes and the expression of proinflammatory HERV-W envelope protein in COVID-19 patients. iScience. 26, 106604. doi: 10.1016/j.isci.2023.106604 37091988 PMC10079620

[B23] Chen L. Deng H. Cui H. Fang J. Zuo Z. Deng J.. (2017). Inflammatory responses and inflammation-associated diseases in organs. Oncotarget. 9, 7204–7218. doi: 10.18632/oncotarget.v9i6 29467962 PMC5805548

[B24] Chen J. Foroozesh M. Qin Z. (2019). Transactivation of human endogenous retroviruses by tumor viruses and their functions in virus-associated Malignancies. Oncogenesis. 8, 6. doi: 10.1038/s41389-018-0114-y 30643113 PMC6331641

[B25] Coffin J. Blomberg J. Fan H. Gifford R. Hatziioannou T. Lindemann D.. (2021). ICTV virus taxonomy profile: retroviridae 2021. J. Gen. Virol. 102, 001712. doi: 10.1099/jgv.0.001712 34939563 PMC8744268

[B26] Compston A. Coles A. (2008). Multiple sclerosis. Lancet 372, 1502–1517. doi: 10.1016/S0140-6736(08)61620-7 18970977

[B27] Contreras-Galindo R. Almodóvar-Camacho S. González-Ramírez S. Lorenzo E. Yamamura Y. (2007a). Short communication: comparative longitudinal studies of HERV-K and HIV-1 RNA titers in HIV-1-infected patients receiving successful versus unsuccessful highly active antiretroviral therapy. AIDS Res. Hum. Retroviruses 23, 1083–1086. doi: 10.1089/aid.2007.0054 17919102

[B28] Contreras-Galindo R. González M. Almodovar-Camacho S. González-Ramírez S. Lorenzo E. Yamamura Y. (2006). A new Real-Time-RT-PCR for quantitation of human endogenous retroviruses type K (HERV-K) RNA load in plasma samples: Increased HERV-K RNA titers in HIV-1 patients with HAART non-suppressive regimens. J. Virol. Methods 136, 51–57. doi: 10.1016/j.jviromet.2006.03.029 16678919

[B29] Contreras-Galindo R. Kaplan M. H. He S. Contreras-Galindo A. C. Gonzalez-Hernandez M. J. Kappes F.. (2013). HIV infection reveals widespread expansion of novel centromeric human endogenous retroviruses. Genome Res. 23, 1505–1513. doi: 10.1101/gr.144303.112 23657884 PMC3759726

[B30] Contreras-Galindo R. Kaplan M. H. Leissner P. Verjat T. Ferlenghi I. Bagnoli F.. (2008). Human endogenous retrovirus K (HML-2) elements in the plasma of people with lymphoma and breast cancer. J. Virol. 82, 9329–9336. doi: 10.1128/JVI.00646-08 18632860 PMC2546968

[B31] Contreras-Galindo R. López P. Vélez R. Yamamura Y. (2007b). HIV-1 infection increases the expression of human endogenous retroviruses type K (HERV-K) in vitro. AIDS Res. Hum. Retroviruses 23, 116–122. doi: 10.1089/aid.2006.0117 17263641

[B32] Dai L. Del Valle L. Miley W. Whitby D. Ochoa A. C. Flemington E. K.. (2018). Transactivation of human endogenous retrovirus K (HERV-K) by KSHV promotes Kaposi’s sarcoma development. Oncogene. 37, 4534–4545. doi: 10.1038/s41388-018-0282-4 29743595 PMC6195842

[B33] Dawson T. Rentia U. Sanford J. Cruchaga C. Kauwe J. S. K. Crandall K. A. (2023). Locus specific endogenous retroviral expression associated with Alzheimer’s disease. Front. Aging Neurosci. 15. doi: 10.3389/fnagi.2023.1186470 PMC10359044 37484691

[B34] de Luca V. Martins Higa A. Malta Romano C. Pimenta Mambrini G. Peroni L. A. Trivinho-Strixino F.. (2019). Cross-reactivity between myelin oligodendrocyte glycoprotein and human endogenous retrovirus W protein: nanotechnological evidence for the potential trigger of multiple sclerosis. Micron. 120, 66–73. doi: 10.1016/j.micron.2019.02.005 30802755

[B35] Dembny P. Newman A. G. Singh M. Hinz M. Szczepek M. Krüger C.. (2020). Human endogenous retrovirus HERV-K(HML-2) RNA causes neurodegeneration through Toll-like receptors. JCI Insight 5 (7). doi: 10.1172/jci.insight.131093 PMC7205273 32271161

[B36] De Meirleir K. L. Khaiboullina S. F. Frémont M. Hulstaert J. Rizvanov A. A. Palotás A.. (2013). Plasmacytoid dendritic cells in the duodenum of individuals diagnosed with myalgic encephalomyelitis are uniquely immunoreactive to antibodies to human endogenous retroviral proteins. Vivo Athens Greece. 27, 177–187. PMC3776582 23422476

[B37] Deng B. Xu W. Wang Z. Liu C. Lin P. Li B.. (2019). An LTR retrotransposon-derived lncRNA interacts with RNF169 to promote homologous recombination. EMBO Rep. 20, e47650. doi: 10.15252/embr.201847650 31486214 PMC6832013

[B38] Denne M. Sauter M. Armbruester V. Licht J. D. Roemer K. Mueller-Lantzsch N. (2007). Physical and functional interactions of human endogenous retrovirus proteins np9 and rec with the promyelocytic leukemia zinc finger protein. J. Virol. 81, 5607–5616. doi: 10.1128/JVI.02771-06 17360752 PMC1900259

[B39] de Parseval N. Casella J. F. Gressin L. Heidmann T. (2001). Characterization of the three HERV-H proviruses with an open envelope reading frame encompassing the immunosuppressive domain and evolutionary history in primates. Virology. 279, 558–569. doi: 10.1006/viro.2000.0737 11162811

[B40] Di Curzio D. Gurm M. Turnbull M. Nadeau M. J. Meek B. Rempel J. D.. (2020). Pro-inflammatory signaling upregulates a neurotoxic conotoxin-like protein encrypted within human endogenous retrovirus-K. Cells. 9, 1584. doi: 10.3390/cells9071584 32629888 PMC7407490

[B41] Douville R. Liu J. Rothstein J. Nath A. (2011). Identification of active loci of a human endogenous retrovirus in neurons of patients with amyotrophic lateral sclerosis. Ann. Neurol. 69, 141–151. doi: 10.1002/ana.22149 21280084 PMC3052883

[B42] Dunn S. J. Martello G. Yordanov B. Emmott S. Smith A. G. (2014). Defining an essential transcription factor program for naïve pluripotency. Science. 344, 1156–1160. doi: 10.1126/science.1248882 24904165 PMC4257066

[B43] Durnaoglu S. Lee S. K. Ahnn J. (2021). Human endogenous retroviruses as gene expression regulators: insights from animal models into human diseases. Mol. Cells 44, 861–878. doi: 10.14348/molcells.2021.5016 34963103 PMC8718366

[B44] Easley R. Van Duyne R. Coley W. Guendel I. Dadgar S. Kehn-Hall K.. (2010). Chromatin dynamics associated with HIV-1 Tat-activated transcription. Biochim. Biophys. Acta BBA - Gene Regul. Mech. 1799, 275–285. doi: 10.1016/j.bbagrm.2009.08.008 PMC2838975 19716452

[B45] Etter M. M. Martins T. A. Kulsvehagen L. Pössnecker E. Duchemin W. Hogan S.. (2022). Severe Neuro-COVID is associated with peripheral immune signatures, autoimmunity and neurodegeneration: a prospective cross-sectional study. Nat. Commun. 13, 6777. doi: 10.1038/s41467-022-34068-0 36351919 PMC9645766

[B46] Feldman E. L. Goutman S. A. Petri S. Mazzini L. Savelieff M. G. Shaw P. J.. (2022). Amyotrophic lateral sclerosis. Lancet 400, 1363–1380. doi: 10.1016/S0140-6736(22)01272-7 36116464 PMC10089700

[B47] Feller W. F. Chopra H. C. (1968). A small virus-like particle observed in human breast cancer by means of electron microscopy2. JNCI J. Natl. Cancer Inst. 40, 1359–1373. doi: 10.1093/jnci/40.6.1359 5660272

[B48] Fevrier B. Vilette D. Archer F. Loew D. Faigle W. Vidal M.. (2004). Cells release prions in association with exosomes. Proc. Natl. Acad. Sci. 101, 9683–9688. doi: 10.1073/pnas.0308413101 15210972 PMC470735

[B49] Franceschi C. Garagnani P. Parini P. Giuliani C. Santoro A. (2018). Inflammaging: a new immune–metabolic viewpoint for age-related diseases. Nat. Rev. Endocrinol. 14, 576–590. doi: 10.1038/s41574-018-0059-4 30046148

[B50] Franceschi C. Salvioli S. Garagnani P. de Eguileor M. Monti D. Capri M. (2017). Immunobiography and the heterogeneity of immune responses in the elderly: A focus on inflammaging and trained immunity. Front. Immunol. 8. doi: 10.3389/fimmu.2017.00982 PMC5559470 28861086

[B51] Frank O. Giehl M. Zheng C. Hehlmann R. Leib-Mösch C. Seifarth W. (2005). Human endogenous retrovirus expression profiles in samples from brains of patients with schizophrenia and bipolar disorders. J. Virol. 79, 10890–10901. doi: 10.1128/JVI.79.17.10890-10901.2005 16103141 PMC1193590

[B52] Franklin G. C. Chretien S. Hanson I. M. Rochefort H. May F. E. Westley B. R. (1988). Expression of human sequences related to those of mouse mammary tumor virus. J. Virol. 62, 1203–1210. doi: 10.1128/jvi.62.4.1203-1210.1988 2831381 PMC253128

[B53] Frasca D. Blomberg B. B. (2016). Inflammaging decreases adaptive and innate immune responses in mice and humans. Biogerontology. 17, 7–19. doi: 10.1007/s10522-015-9578-8 25921609 PMC4626429

[B54] Fülöp T. Dupuis G. Witkowski J. M. Larbi A. (2016). The role of immunosenescence in the development of age-related diseases. Rev Inves Clin. 68, 849–91. 27103044

[B55] Fulop T. Larbi A. Dupuis G. Le Page A. Frost E. H. Cohen A. A.. (2018). Immunosenescence and inflamm-aging as two sides of the same coin: friends or foes? Front. Immunol. 8. doi: 10.3389/fimmu.2017.01960 PMC5767595 29375577

[B56] Fulop T. Larbi A. Pawelec G. Khalil A. Cohen A. A. Hirokawa K.. (2023). Immunology of aging: the birth of inflammaging. Clin. Rev. Allergy Immunol. 64, 109–122. doi: 10.1007/s12016-021-08899-6 34536213 PMC8449217

[B57] Furman D. Campisi J. Verdin E. Carrera-Bastos P. Targ S. Franceschi C.. (2019). Chronic inflammation in the etiology of disease across the life span. Nat. Med. 25, 1822–1832. doi: 10.1038/s41591-019-0675-0 31806905 PMC7147972

[B58] Gabus C. Auxilien S. Péchoux C. Dormont D. Swietnicki W. Morillas M.. (2001a). The prion protein has DNA strand transfer properties similar to retroviral nucleocapsid protein11Edited by J. Karn. J. Mol. Biol. 307, 1011–1021. doi: 10.1006/jmbi.2001.4544 11286552

[B59] Gabus C. Derrington E. Leblanc P. Chnaiderman J. Dormont D. Swietnicki W.. (2001b). The prion protein has RNA binding and chaperoning properties characteristic of nucleocapsid protein NCp7 of HIV-1*. J. Biol. Chem. 276, 19301–19309. doi: 10.1074/jbc.M009754200 11278562

[B60] Garazha A. Ivanova A. Suntsova M. Malakhova G. Roumiantsev S. Zhavoronkov A.. (2015). New bioinformatic tool for quick identification of functionally relevant endogenous retroviral inserts in human genome. Cell Cycle 14, 1476–1484. doi: 10.1080/15384101.2015.1022696 25853282 PMC4612461

[B61] Garcia-Montojo M. Fathi S. Norato G. Smith B. R. Rowe D. B. Kiernan M. C.. (2021). Inhibition of HERV-K (HML-2) in amyotrophic lateral sclerosis patients on antiretroviral therapy. J. Neurol. Sci. 423. doi: 10.1016/j.jns.2021.117358 PMC8009857 33653604

[B62] Garrison K. E. Jones R. B. Meiklejohn D. A. Anwar N. Ndhlovu L. C. Chapman J. M.. (2007). T cell responses to human endogenous retroviruses in HIV-1 infection. PloS Pathog. 3, e165. doi: 10.1371/journal.ppat.0030165 17997601 PMC2065876

[B63] Geis F. K. Goff S. P. (2020). Silencing and transcriptional regulation of endogenous retroviruses: an overview. Viruses. 12, 884. doi: 10.3390/v12080884 32823517 PMC7472088

[B64] Giménez-Orenga K. Martín-Martínez E. Nathanson L. Oltra E. (2023). HERV activation segregates ME/CFS from fibromyalgia and defines a novel nosological entity for patients fulfilling both clinical criteria. bioRxiv. doi: 10.1101/2023.10.05.561025v1

[B65] Göke J. Lu X. Chan Y. S. Ng H. H. Ly L. H. Sachs F.. (2015). Dynamic transcription of distinct classes of endogenous retroviral elements marks specific populations of early human embryonic cells. Cell Stem Cell. 16, 135–141. doi: 10.1016/j.stem.2015.01.005 25658370

[B66] Gold J. Rowe D. B. Kiernan M. C. Vucic S. Mathers S. van Eijk R. P. A.. (2019). Safety and tolerability of Triumeq in amyotrophic lateral sclerosis: the Lighthouse trial. Amyotroph Lateral Scler Front. Degener. 20, 595–604. doi: 10.1080/21678421.2019.1632899 31284774

[B67] Goldsmith D. R. Rapaport M. H. Miller B. J. (2016). A meta-analysis of blood cytokine network alterations in psychiatric patients: comparisons between schizophrenia, bipolar disorder and depression. Mol. Psychiatry 21, 1696–1709. doi: 10.1038/mp.2016.3 26903267 PMC6056174

[B68] Gonzalez-Hernandez M. J. Cavalcoli J. D. Sartor M. A. Contreras-Galindo R. Meng F. Dai M.. (2014). Regulation of the human endogenous retrovirus K (HML-2) transcriptome by the HIV-1 tat protein. J. Virol. 88, 8924–8935. doi: 10.1128/JVI.00556-14 24872592 PMC4136263

[B69] Gonzalez-Hernandez M. J. Swanson M. D. Contreras-Galindo R. Cookinham S. King S. R. Noel R. J.. (2012). Expression of human endogenous retrovirus type K (HML-2) is activated by the tat protein of HIV-1. J. Virol. 86, 7790–7805. doi: 10.1128/JVI.07215-11 22593154 PMC3421662

[B70] Grandi N. Cadeddu M. Blomberg J. Tramontano E. (2016). Contribution of type W human endogenous retroviruses to the human genome: characterization of HERV-W proviral insertions and processed pseudogenes. Retrovirology. 13, 67. doi: 10.1186/s12977-016-0301-x 27613107 PMC5016936

[B71] Greenwood A. D. Vincendeau M. Schmädicke A. C. Montag J. Seifarth W. Motzkus D. (2011). Bovine spongiform encephalopathy infection alters endogenous retrovirus expression in distinct brain regions of cynomolgus macaques (Macaca fascicularis). Mol. Neurodegener. 6, 44. doi: 10.1186/1750-1326-6-44 21699683 PMC3152937

[B72] Groh S. Schotta G. (2017). Silencing of endogenous retroviruses by heterochromatin. Cell Mol. Life Sci. 74, 2055–2065. doi: 10.1007/s00018-017-2454-8 28160052 PMC11107624

[B73] Grow E. J. Flynn R. A. Chavez S. L. Bayless N. L. Wossidlo M. Wesche D. J.. (2015). Intrinsic retroviral reactivation in human preimplantation embryos and pluripotent cells. Nature. 522, 221–225. doi: 10.1038/nature14308 25896322 PMC4503379

[B74] Gruchot J. Herrero F. Weber-Stadlbauer U. Meyer U. Küry P. (2023a). Interplay between activation of endogenous retroviruses and inflammation as common pathogenic mechanism in neurological and psychiatric disorders. Brain Behav. Immun. 107, 242–252. doi: 10.1016/j.bbi.2022.10.007 36270439

[B75] Gruchot J. Kremer D. Küry P. (2019). Neural cell responses upon exposure to human endogenous retroviruses. Front. Genet. 10. doi: 10.3389/fgene.2019.00655/full PMC6637040 31354794

[B76] Gruchot J. Lewen I. Dietrich M. Reiche L. Sindi M. Hecker C.. (2023b). Transgenic expression of the HERV-W envelope protein leads to polarized glial cell populations and a neurodegenerative environment. Proc. Natl. Acad. Sci. 120, e2308187120. doi: 10.1073/pnas.2308187120 37695891 PMC10515160

[B77] Guliyev M. Yilmaz S. Sahin K. Marakli S. Gozukirmizi N. (2013). Human endogenous retrovirus-H insertion screening. Mol. Med. Rep. 7, 1305–1309. doi: 10.3892/mmr.2013.1295 23358623

[B78] Hardy J. A. Higgins G. A. (1992). Alzheimer’s disease: the amyloid cascade hypothesis. Science. 256, 184–185. doi: 10.1126/science.1566067 1566067

[B79] Hashimoto K. Jouhilahti E. M. Töhönen V. Carninci P. Kere J. Katayama S. (2021). Embryonic LTR retrotransposons supply promoter modules to somatic tissues. Genome Res. 31, 1983–1993. doi: 10.1101/gr.275354.121 34675070 PMC8559712

[B80] Hendrickson P. G. Doráis J. A. Grow E. J. Whiddon J. L. Lim J. W. Wike C. L.. (2017). Conserved roles of mouse DUX and human DUX4 in activating cleavage-stage genes and MERVL/HERVL retrotransposons. Nat. Genet. 49, 925–934. doi: 10.1038/ng.3844 28459457 PMC5703070

[B81] Heyne K. Kölsch K. Bruand M. Kremmer E. Grässer F. A. Mayer J.. (2015). Np9, a cellular protein of retroviral ancestry restricted to human, chimpanzee and gorilla, binds and regulates ubiquitin ligase MDM2. Cell Cycle 14, 2619–2633. doi: 10.1080/15384101.2015.1064565 26103464 PMC4614042

[B82] Horssen J.v. Pol S.v. d. Nijland P. Amor S. Perron H. (2016). Human endogenous retrovirus W in brain lesions: Rationale for targeted therapy in multiple sclerosis. Mult Scler Relat. Disord. 8, 11–18. doi: 10.1016/j.msard.2016.04.006 27456869

[B83] Hsiao F. C. Lin M. Tai A. Chen G. Huber B. T. (2006). Cutting edge: epstein-barr virus transactivates the HERV-K18 superantigen by docking to the human complement receptor 2 (CD21) on primary B cells1. J. Immunol. 177, 2056–2060. doi: 10.4049/jimmunol.177.4.2056 16887963

[B84] Hsiao F. C. Tai A. K. Deglon A. Sutkowski N. Longnecker R. Huber B. T. (2009). EBV LMP-2A employs a novel mechanism to transactivate the HERV-K18 superantigen through its ITAM. Virology. 385, 261–266. doi: 10.1016/j.virol.2008.11.025 19070345

[B85] Hurme M. Pawelec G. (2021). Human endogenous retroviruses and ageing. Immun. Ageing. 18, 14. doi: 10.1186/s12979-021-00228-x 33766025 PMC7995696

[B86] Hurst T. P. Magiorkinis G. (2015). Activation of the innate immune response by endogenous retroviruses. J. Gen. Virol. 96, 1207–1218. doi: 10.1099/vir.0.000017 26068187

[B87] Jang S. Lee K. Ju J. H. (2021). Recent updates of diagnosis, pathophysiology, and treatment on osteoarthritis of the knee. Int. J. Mol. Sci. 22, 2619. doi: 10.3390/ijms22052619 33807695 PMC7961389

[B88] Jeong B. H. Jin J. K. Choi E. K. Lee E. Y. Meeker H. C. Kozak C. A.. (2002). Analysis of the expression of endogenous murine leukemia viruses in the brains of senescence-accelerated mice (SAMP8) and the relationship between expression and brain histopathology. J. Neuropathol. Exp. Neurol. 61, 1001–1012. doi: 10.1093/jnen/61.11.1001 12430717

[B89] Jeong B. H. Lee Y. J. Carp R. I. Kim Y. S. (2010). The prevalence of human endogenous retroviruses in cerebrospinal fluids from patients with sporadic Creutzfeldt–Jakob disease. J. Clin. Virol. 47, 136–142. doi: 10.1016/j.jcv.2009.11.016 20005155

[B90] Jern P. Coffin J. M. (2008). Effects of retroviruses on host genome function. Annu. Rev. Genet. 42, 709–732. doi: 10.1146/annurev.genet.42.110807.091501 18694346

[B91] Jern P. Sperber G. O. Blomberg J. (2005). Use of Endogenous Retroviral Sequences (ERVs) and structural markers for retroviral phylogenetic inference and taxonomy. Retrovirology. 2, 50. doi: 10.1186/1742-4690-2-50 16092962 PMC1224870

[B92] Jia H. Huang W. Liu C. Tang S. Zhang J. Chen C.. (2022). Immunosenescence is a therapeutic target for frailty in older adults: a narrative review. Ann. Transl. Med. 10, 1142–1142. doi: 10.21037/atm 36388790 PMC9652526

[B93] Johnston J. B. Silva C. Holden J. Warren K. G. Clark A. W. Power C. (2001). Monocyte activation and differentiation augment human endogenous retrovirus expression: Implications for inflammatory brain diseases. Ann. Neurol. 50, 434–442. doi: 10.1002/ana.1131 11601494

[B94] Jones R. B. Leal F. E. Hasenkrug A. M. Segurado A. C. Nixon D. F. Ostrowski M. A.. (2013). Human Endogenous Retrovirus K(HML-2) Gag and Env specific T-cell responses are not detected in HTLV-I-infected subjects using standard peptide screening methods. J. Negat Results Biomed. 12, 3. doi: 10.1186/1477-5751-12-3 23305161 PMC3560086

[B95] Kämmerer U. Germeyer A. Stengel S. Kapp M. Denner J. (2011). Human endogenous retrovirus K (HERV-K) is expressed in villous and extravillous cytotrophoblast cells of the human placenta. J. Reprod. Immunol. 91, 1–8. doi: 10.1016/j.jri.2011.06.102 21840605

[B96] Kannan S. Chernikova D. Rogozin I. B. Poliakov E. Managadze D. Koonin E. V.. (2015). Transposable element insertions in long intergenic non-coding RNA genes. Front. Bioeng Biotechnol. 3. doi: 10.3389/fbioe.2015.00071 PMC4460805 26106594

[B97] Karamitros T. Paraskevis D. Hatzakis A. Psichogiou M. Elefsiniotis I. Hurst T.. (2016). A contaminant-free assessment of Endogenous Retroviral RNA in human plasma. Sci. Rep. 6, 33598. doi: 10.1038/srep33598 27640347 PMC5027517

[B98] Karlsson H. Bachmann S. Schröder J. McArthur J. Torrey E. F. Yolken R. H. (2001). Retroviral RNA identified in the cerebrospinal fluids and brains of individuals with schizophrenia. Proc. Natl. Acad. Sci. 98, 4634–4639. doi: 10.1073/pnas.061021998 11296294 PMC31886

[B99] Katzourakis A. Pereira V. Tristem M. (2007). Effects of recombination rate on human endogenous retrovirus fixation and persistence. J. Virol. 81, 10712–10717. doi: 10.1128/JVI.00410-07 17634225 PMC2045447

[B100] Kim T. H. Jeon Y. J. Yi J. M. Kim D. S. Huh J. W. Hur C. G.. (2004). The distribution and expression of HERV families in the human genome. Mol. Cells 18, 87–93. doi: 10.1016/S1016-8478(23)13085-8 15359128

[B101] Kolbe A. R. Bendall M. L. Pearson A. T. Paul D. Nixon D. F. Pérez-Losada M.. (2020). Human endogenous retrovirus expression is associated with head and neck cancer and differential survival. Viruses. 12, 956. doi: 10.3390/v12090956 32872377 PMC7552064

[B102] Lachmann R. H. Sadarangani M. Atkinson H. R. Efstathiou S. (1999). An analysis of herpes simplex virus gene expression during latency establishment and reactivation. J. Gen. Virol. 80, 1271–1282. doi: 10.1099/0022-1317-80-5-1271 10355774

[B103] LeBrasseur N. K. Tchkonia T. Kirkland J. L. (2015). Cellular senescence and the biology of aging, disease, and frailty. Nestle Nutr. Inst Workshop Ser. 83, 11–18. doi: 10.1159/000382054 26485647 PMC4780350

[B104] Li Y. Chen Y. Zhang N. Fan D. (2022). Human endogenous retrovirus K (HERV-K) env in neuronal extracellular vesicles: a new biomarker of motor neuron disease. Amyotroph Lateral Scler Front. Degener 23, 100–107. doi: 10.1080/21678421.2021.1936061 34151656

[B105] Li X. Guo Y. Li H. Huang X. Pei Z. Wang X.. (2021). Infection by diverse HIV-1 subtypes leads to different elevations in HERV-K transcriptional levels in human T cell lines. Front. Microbiol. 12. doi: 10.3389/fmicb.2021.662573 PMC8165174 34079529

[B106] Li W. Lee M. H. Henderson L. Tyagi R. Bachani M. Steiner J.. (2015). Human endogenous retrovirus-K contributes to motor neuron disease. Sci. Transl. Med. 7, 307ra153. doi: 10.1126/scitranslmed.aac8201 PMC6344353 26424568

[B107] Li J. Lin J. Lin J. R. Farris M. Robbins L. Andrada L.. (2022). Dolutegravir inhibits proliferation and motility of BT-20 tumor cells through inhibition of human endogenous retrovirus type K. Cureus 14, e26525. doi: 10.7759/cureus.26525 35936147 PMC9345775

[B108] Li F. Nellåker C. Sabunciyan S. Yolken R. H. Jones-Brando L. Johansson A. S.. (2014). Transcriptional derepression of the ERVWE1 locus following influenza A virus infection. J. Virol. 88, 4328–4337. doi: 10.1128/JVI.03628-13 24478419 PMC3993755

[B109] Liu X. Liu Z. Wu Z. Ren J. Fan Y. Sun L.. (2023). Resurrection of endogenous retroviruses during aging reinforces senescence. Cell. 186, 287–304.e26. doi: 10.1016/j.cell.2022.12.017 36610399

[B110] Lowe C. B. Bejerano G. Haussler D. (2007). Thousands of human mobile element fragments undergo strong purifying selection near developmental genes. Proc. Natl. Acad. Sci. 104, 8005–8010. doi: 10.1073/pnas.0611223104 17463089 PMC1876562

[B111] Löwer R. Löwer J. Frank H. Harzmann R. Kurth R. (1984). Human teratocarcinomas cultured in *vitro* produce unique retrovirus-like viruses. J. Gen. Virol. 65, 887–898. doi: 10.1099/0022-1317-65-5-887 6202829

[B112] Lu X. Sachs F. Ramsay L. Jacques PÉ Göke J. Bourque G.. (2014). The retrovirus HERVH is a long noncoding RNA required for human embryonic stem cell identity. Nat. Struct. Mol. Biol. 21, 423–425. doi: 10.1038/nsmb.2799 24681886

[B113] Macfarlan T. S. Gifford W. D. Driscoll S. Lettieri K. Rowe H. M. Bonanomi D.. (2012). Embryonic stem cell potency fluctuates with endogenous retrovirus activity. Nature. 487, 57–63. doi: 10.1038/nature11244 22722858 PMC3395470

[B114] Mameli G. Astone V. Khalili K. Serra C. Sawaya B. E. Dolei A. (2007). Regulation of the syncytin-1 promoter in human astrocytes by multiple sclerosis-related cytokines. Virology. 362, 120–130. doi: 10.1016/j.virol.2006.12.019 17258784

[B115] Mameli G. Poddighe L. Mei A. Uleri E. Sotgiu S. Serra C.. (2012). Expression and activation by epstein barr virus of human endogenous retroviruses-W in blood cells and astrocytes: inference for multiple sclerosis. PloS One 7, e44991. doi: 10.1371/journal.pone.0044991 23028727 PMC3459916

[B116] Manca M. A. Solinas T. Simula E. R. Noli M. Ruberto S. Madonia M.. (2022). HERV-K and HERV-H env proteins induce a humoral response in prostate cancer patients. Pathogens. 11, 95. doi: 10.3390/pathogens11010095 35056043 PMC8778306

[B117] Mao J. Zhang Q. Cong Y. S. (2021). Human endogenous retroviruses in development and disease. Comput. Struct. Biotechnol. J. 19, 5978–5986. doi: 10.1016/j.csbj.2021.10.037 34849202 PMC8604659

[B118] Marston J. L. Greenig M. Singh M. Bendall M. L. Duarte R. R. R. Feschotte C.. (2021). SARS-CoV-2 infection mediates differential expression of human endogenous retroviruses and long interspersed nuclear elements. JCI Insight 6 (24). doi: 10.1172/jci.insight.147170 PMC8783694 34731091

[B119] Matos A de M. B. Dahy F. E. de Moura J. V. L. Marcusso R. M. N. Gomes A. B. F. Carvalho F. M. M.. (2021). Subacute cognitive impairment in individuals with mild and moderate COVID-19: A case series. Front. Neurol. 12. doi: 10.3389/fneur.2021.678924 PMC8371908 34421788

[B120] Mattson M. P. (2004). Pathways towards and away from Alzheimer’s disease. Nature. 430, 631–639. doi: 10.1038/nature02621 15295589 PMC3091392

[B121] Mercer T. R. Dinger M. E. Mattick J. S. (2009). Long non-coding RNAs: insights into functions. Nat. Rev. Genet. 10, 155–159. doi: 10.1038/nrg2521 19188922

[B122] Mi S. Lee X. Li X.p. Veldman G. M. Finnerty H. Racie L.. (2000). Syncytin is a captive retroviral envelope protein involved in human placental morphogenesis. Nature. 403, 785–789. doi: 10.1038/35001608 10693809

[B123] Michaud H. A. de Mulder M. SenGupta D. Deeks S. G. Martin J. N. Pilcher C. D.. (2014). Trans-activation, post-transcriptional maturation, and induction of antibodies to HERV-K (HML-2) envelope transmembrane protein in HIV-1 infection. Retrovirology. 11, 10. doi: 10.1186/1742-4690-11-10 24472118 PMC3907665

[B124] Morris G. Maes M. Murdjeva M. Puri B. K. (2019). Do human endogenous retroviruses contribute to multiple sclerosis, and if so, how? Mol. Neurobiol. 56, 2590–2605. doi: 10.1007/s12035-018-1255-x 30047100 PMC6459794

[B125] Nali L. H. Olival G. S. Montenegro H. Silva I. T. da Dias-Neto E. Naya H.. (2022). Human endogenous retrovirus and multiple sclerosis: A review and transcriptome findings. Mult Scler Relat. Disord. 57. doi: 10.1016/j.msard.2021.103383 34922254

[B126] Nali L. H. S. Oliveira A. C. S. Alves D. O. Caleiro G. S. Nunes C. F. Gerhardt D.. (2017). Expression of human endogenous retrovirus K and W in babies. Arch. Virol. 162, 857–861. doi: 10.1007/s00705-016-3167-2 27885560

[B127] Nellåker C. Yao Y. Jones-Brando L. Mallet F. Yolken R. H. Karlsson H. (2006). Transactivation of elements in the human endogenous retrovirus W family by viral infection. Retrovirology. 3, 44. doi: 10.1186/1742-4690-3-44 16822326 PMC1539011

[B128] Nelson P. Davari-Ejtehadi H. Nevill A. Bowman S. (2010). Endogenous retrovirus erv-3 is not implicated in rheumatoid arthritis but may provide a biomarker for osteoarthritis. J. Rheumatol. 37, 473–473. doi: 10.3899/jrheum.090735 20147487

[B129] Nevalainen T. Autio A. Mishra B. H. Marttila S. Jylhä M. Hurme M. (2018). Aging-associated patterns in the expression of human endogenous retroviruses. PloS One 13, e0207407. doi: 10.1371/journal.pone.0207407 30513106 PMC6279030

[B130] Nisole S. Saïb A. (2004). Early steps of retrovirus replicative cycle. Retrovirology. 1, 9. doi: 10.1186/1742-4690-1-9 15169567 PMC421752

[B131] Noorali S. Rotar I. C. Lewis C. Pestaner J. P. Pace D. G. Sison A.. (2009). Role of HERV-W syncytin-1 in placentation and maintenance of human pregnancy. Appl. Immunohistochem Mol. Morphol. 17, 319. doi: 10.1097/PAI.0b013e31819640f9 19407656

[B132] Nurk S. Koren S. Rhie A. Rautiainen M. Bzikadze A. V. Mikheenko A.. (2022). The complete sequence of a human genome. Science. 376, 44–53. doi: 10.1126/science.abj6987 35357919 PMC9186530

[B133] Ohnuki M. Tanabe K. Sutou K. Teramoto I. Sawamura Y. Narita M.. (2014). Dynamic regulation of human endogenous retroviruses mediates factor-induced reprogramming and differentiation potential. Proc. Natl. Acad. Sci. 111, 12426–12431. doi: 10.1073/pnas.1413299111 25097266 PMC4151758

[B134] Olival G. Faria T. Nali L. H. de Oliveira A. C. Casseb J. Vidal J. E.. (2013). Genomic analysis of ERVWE2 locus in patients with multiple sclerosis: absence of genetic association but potential role of human endogenous retrovirus type W elements in molecular mimicry with myelin antigen. Front. Microbiol. 4. doi: 10.3389/fmicb.2013.00172 PMC3693062 23805135

[B135] Ono M. Yasunaga T. Miyata T. Ushikubo H. (1986). Nucleotide sequence of human endogenous retrovirus genome related to the mouse mammary tumor virus genome. J. Virol. 60, 589–598. doi: 10.1128/jvi.60.2.589-598.1986 3021993 PMC288930

[B136] Ovejero T. Sadones O. Sánchez-Fito T. Almenar-Pérez E. Espejo J. A. Martín-Martínez E.. (2020). Activation of transposable elements in immune cells of fibromyalgia patients. Int. J. Mol. Sci. 21, 1366. doi: 10.3390/ijms21041366 32085571 PMC7072917

[B137] Paces J. (2004). HERVd: the human endogenous retroViruses database: update. Nucleic Acids Res. 32, 50D–5 50. doi: 10.1093/nar/gkh075 PMC308809 14681356

[B138] Pérot P. Mullins C. S. Naville M. Bressan C. Hühns M. Gock M.. (2015). Expression of young HERV-H loci in the course of colorectal carcinoma and correlation with molecular subtypes. Oncotarget. 6, 40095–40111. doi: 10.18632/oncotarget.v6i37 26517682 PMC4741882

[B139] Perron H. Dougier-Reynaud H. L. Lomparski C. Popa I. Firouzi R. Bertrand J. B.. (2013). Human endogenous retrovirus protein activates innate immunity and promotes experimental allergic encephalomyelitis in mice. PloS One 8, e80128. doi: 10.1371/journal.pone.0080128 24324591 PMC3855614

[B140] Perron H. Garson J. A. Bedin F. Beseme F. Paranhos-Baccala G. Komurian-Pradel F.. (1997). Molecular identification of a novel retrovirus repeatedly isolated from patients with multiple sclerosis. Proc. Natl. Acad. Sci. 94, 7583–7588. doi: 10.1073/pnas.94.14.7583 9207135 PMC23865

[B141] Perron H. Hamdani N. Faucard R. Lajnef M. Jamain S. Daban-Huard C.. (2012). Molecular characteristics of Human Endogenous Retrovirus type-W in schizophrenia and bipolar disorder. Transl. Psychiatry 2, e201. doi: 10.1038/tp.2012.125 23212585 PMC3565190

[B142] Perron H. Lazarini F. Ruprecht K. Péchoux-Longin C. Seilhean D. Sazdovitch V.. (2005). Human endogenous retrovirus (HERV)-W ENV and GAG proteins: Physiological expression in human brain and pathophysiological modulation in multiple sclerosis lesions. J. Neurovirol. 11, 23–33. doi: 10.1080/13550280590901741 15804956

[B143] Perzova R. Graziano E. Sanghi S. Welch C. Benz P. Abbott L.. (2013). Increased seroreactivity to HERV-K10 peptides in patients with HTLV myelopathy. Virol. J. 10, 360. doi: 10.1186/1743-422X-10-360 24365054 PMC3878045

[B144] Poiesz B. J. Ruscetti F. W. Gazdar A. F. Bunn P. A. Minna J. D. Gallo R. C. (1980). Detection and isolation of type C retrovirus particles from fresh and cultured lymphocytes of a patient with cutaneous T-cell lymphoma. Proc. Natl. Acad. Sci. 77, 7415–7419. doi: 10.1073/pnas.77.12.7415 6261256 PMC350514

[B145] Poole B. D. Scofield R. H. Harley J. B. James J. A. (2006). Epstein-Barr virus and molecular mimicry in systemic lupus erythematosus. Autoimmunity. 39, 63–70. doi: 10.1080/08916930500484849 16455583

[B146] Prion Biology and Diseases. (2024). Available online at: https://www.cshlpress.com/default.tpl?cart=1706321086628263598&fromlink=T&linkaction=full&linksortby=oop_title&–eqSKUdatarq=454.

[B147] Ramasamy R. Joseph B. Whittall T. (2017). Potential molecular mimicry between the human endogenous retrovirus W family envelope proteins and myelin proteins in multiple sclerosis. Immunol. Lett. 183, 79–85. doi: 10.1016/j.imlet.2017.02.003 28189601

[B148] Rangel S. C. da Silva M. D. da Silva A. L. dos Santos J de M. B. Neves L. M. Pedrosa A.. (2022). Human endogenous retroviruses and the inflammatory response: A vicious circle associated with health and illness. Front. Immunol. 13. doi: 10.3389/fimmu.2022.1057791 PMC9744114 36518758

[B149] Rodrigues L. S. da Silva Nali L. H. Leal C. O. D. Sabino E. C. Lacerda E. M. Kingdon C. C.. (2019). HERV-K and HERV-W transcriptional activity in myalgic encephalomyelitis/chronic fatigue syndrome. Autoimmun Highlights. 10, 12. doi: 10.1186/s13317-019-0122-8 PMC7065355 32257068

[B150] Rolland A. Jouvin-Marche E. Saresella M. Ferrante P. Cavaretta R. Créange A.. (2005). Correlation between disease severity and in *vitro* cytokine production mediated by MSRV (Multiple Sclerosis associated RetroViral element) envelope protein in patients with multiple sclerosis. J. Neuroimmunol. 160, 195–203. doi: 10.1016/j.jneuroim.2004.10.019 15710473

[B151] Rolland A. Jouvin-Marche E. Viret C. Faure M. Perron H. Marche P. N. (2006). The envelope protein of a human endogenous retrovirus-W family activates innate immunity through CD14/TLR4 and promotes th1-like responses1. J. Immunol. 176, 7636–7644. doi: 10.4049/jimmunol.176.12.7636 16751411

[B152] Santoni F. A. Guerra J. Luban J. (2012). HERV-H RNA is abundant in human embryonic stem cells and a precise marker for pluripotency. Retrovirology. 9, 111. doi: 10.1186/1742-4690-9-111 23253934 PMC3558390

[B153] Sarngadharan M. G. Sarin P. S. Reitz M. S. Gallo R. C. (1972). Reverse transcriptase activity of human acute leukemic cells: purification of the enzyme, response to AMV 70S RNA, and characterization of the DNA product. Nat. New Biol. 240, 67–72. doi: 10.1038/newbio240067a0 4117890

[B154] Sauter M. Schommer S. Kremmer E. Remberger K. Dölken G. Lemm I.. (1995). Human endogenous retrovirus K10: expression of Gag protein and detection of antibodies in patients with seminomas. J. Virol. 69, 414–421. doi: 10.1128/jvi.69.1.414-421.1995 7983737 PMC188589

[B155] Siebenthall K. T. Miller C. P. Vierstra J. D. Mathieu J. Tretiakova M. Reynolds A.. (2019). Integrated epigenomic profiling reveals endogenous retrovirus reactivation in renal cell carcinoma. eBioMedicine. 41, 427–442. doi: 10.1016/j.ebiom.2019.01.063 30827930 PMC6441874

[B156] Slokar G. Hasler G. (2015). Human endogenous retroviruses as pathogenic factors in the development of schizophrenia. Front. Psychiatry 6. doi: 10.3389/fpsyt.2015.00183 PMC4707225 26793126

[B157] Soygur B. Moore H. (2016). Expression of Syncytin 1 (HERV-W), in the preimplantation human blastocyst, embryonic stem cells and trophoblast cells derived in *vitro* . Hum. Reprod. 31, 1455–1461. doi: 10.1093/humrep/dew097 27173892

[B158] Soygur B. Sati L. (2016). The role of syncytins in human reproduction and reproductive organ cancers. Reproduction 152, R167–R178. doi: 10.1530/REP-16-0031 27486264

[B159] Steiner J. P. Bachani M. Malik N. DeMarino C. Li W. Sampson K.. (2022). Human endogenous retrovirus K envelope in spinal fluid of amyotrophic lateral sclerosis is toxic. Ann. Neurol. 92, 545–561. doi: 10.1002/ana.26452 35801347 PMC9489628

[B160] Strawbridge R. Sartor M. L. Scott F. Cleare A. J. (2019). Inflammatory proteins are altered in chronic fatigue syndrome-A systematic review and meta-analysis. Neurosci. Biobehav. Rev. 107, 69–83. doi: 10.1016/j.neubiorev.2019.08.011 31465778

[B161] Stricker E. Peckham-Gregory E. C. Scheurer M. E. (2023a). CancerHERVdb: human endogenous retrovirus (HERV) expression database for human cancer accelerates studies of the retrovirome and predictions for HERV-based therapies. J. Virol. 97, e00059–e00023. doi: 10.1128/jvi.00059-23 37255431 PMC10308937

[B162] Stricker E. Peckham-Gregory E. C. Scheurer M. E. (2023b). HERVs and cancer—A comprehensive review of the relationship of human endogenous retroviruses and human cancers. Biomedicines. 11, 936. doi: 10.3390/biomedicines11030936 36979914 PMC10046157

[B163] Subramanian R. P. Wildschutte J. H. Russo C. Coffin J. M. (2011). Identification, characterization, and comparative genomic distribution of the HERV-K (HML-2) group of human endogenous retroviruses. Retrovirology. 8, 90. doi: 10.1186/1742-4690-8-90 22067224 PMC3228705

[B164] Sutkowski N. Chen G. Calderon G. Huber B. T. (2004). Epstein-barr virus latent membrane protein LMP-2A is sufficient for transactivation of the human endogenous retrovirus HERV-K18 superantigen. J. Virol. 78, 7852–7860. doi: 10.1128/JVI.78.14.7852-7860.2004 15220463 PMC434102

[B165] Sutkowski N. Conrad B. Thorley-Lawson D. A. Huber B. T. (2001). Epstein-barr virus transactivates the human endogenous retrovirus HERV-K18 that encodes a superantigen. Immunity. 15, 579–589. doi: 10.1016/S1074-7613(01)00210-2 11672540

[B166] Tamouza R. Meyer U. Foiselle M. Richard J. R. Wu C. L. Boukouaci W.. (2021). Identification of inflammatory subgroups of schizophrenia and bipolar disorder patients with HERV-W ENV antigenemia by unsupervised cluster analysis. Transl. Psychiatry 11, 1–8. doi: 10.1038/s41398-021-01499-0 34230451 PMC8260666

[B167] Tanajura D. Castro N. Oliveira P. Neto A. Muniz A. Carvalho N. B.. (2015). Neurological manifestations in human T-cell lymphotropic virus type 1 (HTLV-1)–infected individuals without HTLV-1–associated myelopathy/tropical spastic paraparesis: A longitudinal cohort study. Clin. Infect. Dis. 61, 49–56. doi: 10.1093/cid/civ229 25820277 PMC4542911

[B168] Tansey M. G. Wallings R. L. Houser M. C. Herrick M. K. Keating C. E. Joers V. (2022). Inflammation and immune dysfunction in Parkinson disease. Nat. Rev. Immunol. 22, 657–673. doi: 10.1038/s41577-022-00684-6 35246670 PMC8895080

[B169] Toufaily C. Landry S. Leib-Mosch C. Rassart E. Barbeau B. (2011). Activation of LTRs from different human endogenous retrovirus (HERV) families by the HTLV-1 tax protein and T-cell activators. Viruses. 3, 2146–2159. doi: 10.3390/v3112146 22163338 PMC3230845

[B170] Tristem M. (2000). Identification and characterization of novel human endogenous retrovirus families by phylogenetic screening of the human genome mapping project database. J. Virol. 74, 3715–3730. doi: 10.1128/JVI.74.8.3715-3730.2000 10729147 PMC111881

[B171] Vargiu L. Rodriguez-Tomé P. Sperber G. O. Cadeddu M. Grandi N. Blikstad V.. (2016). Classification and characterization of human endogenous retroviruses; mosaic forms are common. Retrovirology. 13, 7. doi: 10.1186/s12977-015-0232-y 26800882 PMC4724089

[B172] Vastenhouw N. L. Cao W. X. Lipshitz H. D. (2019). The maternal-to-zygotic transition revisited. Development 146, dev161471. doi: 10.1242/dev.161471 31189646

[B173] Vincendeau M. Göttesdorfer I. Schreml J. M. H. Wetie A. G. N. Mayer J. Greenwood A. D.. (2015). Modulation of human endogenous retrovirus (HERV) transcription during persistent and *de novo* HIV-1 infection. Retrovirology. 12, 27. doi: 10.1186/s12977-015-0156-6 25886562 PMC4375885

[B174] Vojtechova I. Machacek T. Kristofikova Z. Stuchlik A. Petrasek T. (2022). Infectious origin of Alzheimer’s disease: Amyloid beta as a component of brain antimicrobial immunity. PloS Pathog. 18, e1010929. doi: 10.1371/journal.ppat.1010929 36395147 PMC9671327

[B175] Volkman H. E. Stetson D. B. (2014). The enemy within: endogenous retroelements and autoimmune disease. Nat. Immunol. 15, 415–422. doi: 10.1038/ni.2872 24747712 PMC4131434

[B176] Voon H. P. J. Gibbons R. J. (2016). Maintaining memory of silencing at imprinted differentially methylated regions. Cell Mol. Life Sci. 73, 1871–1879. doi: 10.1007/s00018-016-2157-6 26883803 PMC4819931

[B177] Wahl A. Al-Harthi L. (2023). HIV infection of non-classical cells in the brain. Retrovirology. 20, 1. doi: 10.1186/s12977-023-00616-9 36639783 PMC9840342

[B178] Wallace A. D. Wendt G. A. de Smith A. J. Wiemels J. L. Francis S. S. (2018). To ERV is human: A phenotype-wide scan linking polymorphic human endogenous retrovirus-K insertions to complex phenotypes. Front. Genet. 9. doi: 10.3389/fgene.2018.00298/full PMC6102640 30154825

[B179] Wang H. An Z. Liu Y. Ding G. Deng G. Ji C. (2024). Clinical significance of HHLA2 as a novel therapeutic target for colorectal cancer. Curr. Cancer Drug Targets 24, 1–10. doi: 10.2174/0115680096290709240102064645 38299402

[B180] Wang Y. Holland J. F. Bleiweiss I. J. Melana S. Liu X. Pelisson L.. (1995). Detection of mammary tumor virus ENV gene-like sequences in human breast cancer’. Cancer Res. 55 (22), 5173-5179. 7585568

[B181] Wang J. Xie G. Singh M. Ghanbarian A. T. Raskó T. Szvetnik A.. (2014). Primate-specific endogenous retrovirus-driven transcription defines naive-like stem cells. Nature. 516, 405–409. doi: 10.1038/nature13804 25317556

[B182] Wang T. Zeng J. Lowe C. B. Sellers R. G. Salama S. R. Yang M.. (2007). Species-specific endogenous retroviruses shape the transcriptional network of the human tumor suppressor protein p53. Proc. Natl. Acad. Sci. 104, 18613–18618. doi: 10.1073/pnas.0703637104 18003932 PMC2141825

[B183] Wieland L. Schwarz T. Engel K. Volkmer I. Krüger A. Tarabuko A.. (2022). Epstein-barr virus-induced genes and endogenous retroviruses in immortalized B cells from patients with multiple sclerosis. Cells. 11, 3619. doi: 10.3390/cells11223619 36429047 PMC9688211

[B184] Wildschutte J. H. Williams Z. H. Montesion M. Subramanian R. P. Kidd J. M. Coffin J. M. (2016). Discovery of unfixed endogenous retrovirus insertions in diverse human populations. Proc. Natl. Acad. Sci. 113, E2326–E2334. doi: 10.1073/pnas.1602336113 27001843 PMC4843416

[B185] Wolfe F. Clauw D. J. Fitzcharles M. A. Goldenberg D. L. Häuser W. Katz R. S.. (2011). Fibromyalgia criteria and severity scales for clinical and epidemiological studies: A modification of the ACR preliminary diagnostic criteria for fibromyalgia. J. Rheumatol. 38, 1113–1122. doi: 10.3899/jrheum.100594 21285161

[B186] Yao M. Wang S. Han Y. Zhao H. Yin Y. Zhang Y.. (2023). Micro-inflammation related gene signatures are associated with clinical features and immune status of fibromyalgia. J. Transl. Med. 21, 594. doi: 10.1186/s12967-023-04477-w 37670381 PMC10478377

[B187] Yu H. Robertson E. S. (2023). Epstein–barr virus history and pathogenesis. Viruses. 15, 714. doi: 10.3390/v15030714 36992423 PMC10056551

[B188] Zanrè V. Bellinato F. Cardile A. Passarini C. Monticelli J. Di Bella S.. (2024). Lamivudine, doravirine, and cabotegravir downregulate the expression of human endogenous retroviruses (HERVs), inhibit cell growth, and reduce invasive capability in melanoma cell lines. Int. J. Mol. Sci. 25, 1615. doi: 10.3390/ijms25031615 38338893 PMC10855363

[B189] Zhang M. Zheng S. Liang J. Q. (2022). Transcriptional and reverse transcriptional regulation of host genes by human endogenous retroviruses in cancers. Front. Microbiol. 13. doi: 10.3389/fmicb.2022.946296 PMC9343867 35928153

[B190] Zhou Y. Hong Y. Huang H. (2016). Triptolide attenuates inflammatory response in membranous glomerulo-nephritis rat via downregulation of NF-κB signaling pathway. Kidney Blood Press Res. 41, 901–910. doi: 10.1159/000452591 27871079

[B191] Zhou S. Liu L. Lu X. (2023). Endogenous retroviruses make aging go viral. Life Med. 2, lnad001. doi: 10.1093/lifemedi/lnad001 PMC11749615 39872956

